# Non-ventilator-associated ICU-acquired pneumonia (NV-ICU-AP) in patients with acute exacerbation of COPD: From the French OUTCOMEREA cohort

**DOI:** 10.1186/s13054-023-04631-2

**Published:** 2023-09-19

**Authors:** Louis-Marie Galerneau, Sébastien Bailly, Nicolas Terzi, Stéphane Ruckly, Maité Garrouste-Orgeas, Johanna Oziel, Vivien Hong Tuan Ha, Marc Gainnier, Shidasp Siami, Claire Dupuis, Jean-Marie Forel, Anaïs Dartevel, Julien Dessajan, Christophe Adrie, Dany Goldgran-Toledano, Virginie Laurent, Laurent Argaud, Jean Reignier, Jean-Louis Pepin, Michael Darmon, Jean-François Timsit, OUTCOME R. E. A. network, Jean-François Timsit, Elie Azoulay, Maïté Garrouste-Orgeas, Jean-Ralph Zahar, Bruno Mourvillier, Michael Darmon, Corinne Alberti, Stephane Ruckly, Sébastien Bailly, Aurélien Vannieuwenhuyze, Christophe Adrie, Carole Agasse, Bernard Allaouchiche, Olivier Andremont, Pascal Andreu, Laurent Argaud, Claire Ara-Somohano, Elie Azoulay, Francois Barbier, Jean-Pierre Bedos, Thomas Baudry, Julien Bohé, Lila Bouadma, Jeremy Bourenne, Noel Brule, Frank Chemouni, Julien Carvelli Elisabeth Coupez, Michael Darmon, Claire Dupuis, Etienne de Montmollin, Loa Dopeux, Anne-Sylvie Dumenil, Claire Dupuis, Jean-Marc Forel, Marc Gainnier, Charlotte Garret, Dany Goldgran-Tonedano, Steven Grangé, Antoine Gros, Hédia Hammed, Akim Haouache, Tarik Hissem, Vivien Hong Tuan Ha, Sébastien Jochmans, Jean-Baptiste Joffredo, Hatem Kallel, Guillaume Lacave, Virgine Laurent, Alexandre Lautrette, Clément Le bihan, Virgine Lemiale, David Luis, Guillaume Marcotte, Jordane Lebut, Bruno Mourvillier, Benoît Misset, Bruno Mourvillier, Mathild Neuville, Laurent Nicolet, Johanna Oziel, Laurent Papazian, Juliette Patrier, Benjamin Planquette, Aguila Radjou, Marie Simon, Romain Sonneville, Jean Reignier, Bertrand Souweine, Carole Schwebel, Shidasp Siami, Romain Sonneville, Nicolas Terzi, Gilles Troché, Fabrice Thiollieres, Guillaume Thierry, Guillaume Van Der Meersch, Marion Venot, Florent Wallet, Sondes Yaacoubi, Olivier Zambon, Jonathan Zarka, Mireille Adda, Vanessa Vindrieux, Marion Provent, Sylvie de la Salle, Pauline Enguerrand, Vincent Gobert, Stéphane Guessens, Helene Merle, Nadira Kaddour, Boris Berthe, Samir Bekkhouche, Kaouttar Mellouk, Mélaine Lebrazic, Carole Ouisse, Diane Maugars, Christelle Aparicio, Igor Theodose, Manal Nouacer, Veronique Deiler, Fariza Lamara, Myriam Moussa, Atika Mouaci, Nassima Viguier

**Affiliations:** 1 Medical Intensive Care Unit, University Hospital of Grenoble Alpes, 10217 38043 Grenoble, CS France; 2 Grenoble Alpes University, INSERM 1300, HP2 Grenoble, France; 3 Department of Biostatistics, Outcomerea, Paris, France; 4 Medical Unit, French and British Hospital Cognacq-Jay Fondation, Levallois-Perret, France; 5 Intensive Care Unit, Avicenne Hospital, AP-HP, Paris, France; 6 Medical Intensive Care Unit, Meaux Hospital, Meaux, France; 7 Medical Intensive Care Unit, La Timone Hospital, Marseille, France; 8 Critical Care Medicine Unit, Etampes-Dourdan Hospital, Etampes, France; 9 Medical Intensive Care Unit, Gabriel Montpied University Hospital, Clermont-Ferrand, France; 10 Medical Intensive Care Unit, Nord University Hospital, Marseille, France; 11 Medical and Infectious Diseases Intensive Care Unit (MI2), Bichat Hospital, AP-HP, Paris, France; 12 Polyvalent Intensive Care Unit, Delafontaine Hospital, Saint-Denis, France; 13 Medical Intensive Care Unit, Le Raincy-Montfermeil Hospital, Montfermeil, France; 14 Intensive Care Unit, André Mignot Hospital, Le Chesnay, France; 15 Medical Intensive Care Unit, Edouard Herriot Hospital, Lyon Civil Hospices, Lyon, France; 16 Medical Intensive Care Unit, Nantes University Hospital, Nantes, France; 17 Intensive Care Unit, Saint-Louis Hospital, AP-HP, Paris, France

**Keywords:** Intensive care medicine, Non-ventilator-associated ICU-acquired pneumonia, Acute exacerbation of chronic obstructive pulmonary disease, Prevalence, Prognosis

## Abstract

**Background:**

Non-ventilator-associated ICU-acquired pneumonia (NV-ICU-AP), a nosocomial pneumonia that is not related to invasive mechanical ventilation (IMV), has been less studied than ventilator-associated pneumonia, and never in the context of patients in an ICU for severe acute exacerbation of chronic obstructive pulmonary disease (AECOPD), a common cause of ICU admission. This study aimed to determine the factors associated with NV-ICU-AP occurrence and assess the association between NV-ICU-AP and the outcomes of these patients.

**Methods:**

Data were extracted from the French ICU database, OutcomeRea™. Using survival analyses with competing risk management, we sought the factors associated with the occurrence of NV-ICU-AP. Then we assessed the association between NV-ICU-AP and mortality, intubation rates, and length of stay in the ICU.

**Results:**

Of the 844 COPD exacerbations managed in ICUs without immediate IMV, NV-ICU-AP occurred in 42 patients (5%) with an incidence density of 10.8 per 1,000 patient-days. In multivariate analysis, prescription of antibiotics at ICU admission (sHR, 0.45 [0.23; 0.86], p = 0.02) and no decrease in consciousness (sHR, 0.35 [0.16; 0.76]; p < 0.01) were associated with a lower risk of NV-ICU-AP. After adjusting for confounders, NV-ICU-AP was associated with increased 28-day mortality (HR = 3.03 [1.36; 6.73]; p < 0.01), an increased risk of intubation (csHR, 5.00 [2.54; 9.85]; p < 0.01) and with a 10-day increase in ICU length of stay (p < 0.01).

**Conclusion:**

We found that NV-ICU-AP incidence reached 10.8/1000 patient-days and was associated with increased risks of intubation, 28-day mortality, and longer stay for patients admitted with AECOPD.

**Supplementary Information:**

The online version contains supplementary material available at 10.1186/s13054-023-04631-2.

## Introduction

Severe acute exacerbation of COPD (AECOPD) is a common cause of ICU admission [1] and nosocomial pneumonia is the most frequently reported nosocomial infection in intensive care units (ICUs) [2, 3]. While ventilator-associated pneumonia (VAP) acquired in ICUs has been widely studied [4], there are less consistent data on nosocomial ICU-acquired pneumonia in patients without invasive mechanical ventilation (IMV) (Non-ventilator-associated ICU-acquired pneumonia (NV-ICU-AP)) [5–9].

Non-ventilator hospital-acquired pneumonia (NV-HAP) in or outside the ICU, is associated with a similar or higher risk of mortality than VAP, significant morbidity, and high associated costs [8–14], but NV-HAPs are currently less tracked, reported, and prevented than VAPs [15, 16]. Nevertheless, a growing interest in NV-ICU-APs has recently been observed, and as remarked by *Vallecoccia *et al*.*, nosocomial pneumonia is a multifaced disease with NV-ICU-AP being one of them [7–9, 16, 17]. As pointed out by *Bergin *et al*.,* evaluating the risks of NV-ICU-AP and its outcomes is essential to identify those patients at the highest risk of and from NV-ICU-AP. In addition, studies are needed on diagnostic criteria, new treatments, and prevention strategies focused on the patients who are most likely to benefit [16].

Studies of hospitalised patients suggest that the prevalence of NV-HAP is high in patients with COPD [18–21]. However, NV-ICU-APs have been poorly studied in COPD patients [22, 23] and never in patients admitted to an ICU for severe AECOPD.

NV-ICU-AP is a major concern in patients with COPD exacerbation because of its potentially adverse impact in terms of medical and/or economic burden; particularly in the context of reducing the need for invasive mechanical ventilation and switching to management using non-invasive ventilation (NIV) [24].

The aim of this study was to investigate, using the prospective French OutcomeRea™ database, the factors associated with the occurrence of NV-ICU-AP in ICU patients with severe AECOPD and the association between NV-ICU-AP and the outcomes of these patients.

## METHODS

### Study population

**Fig. 1:**
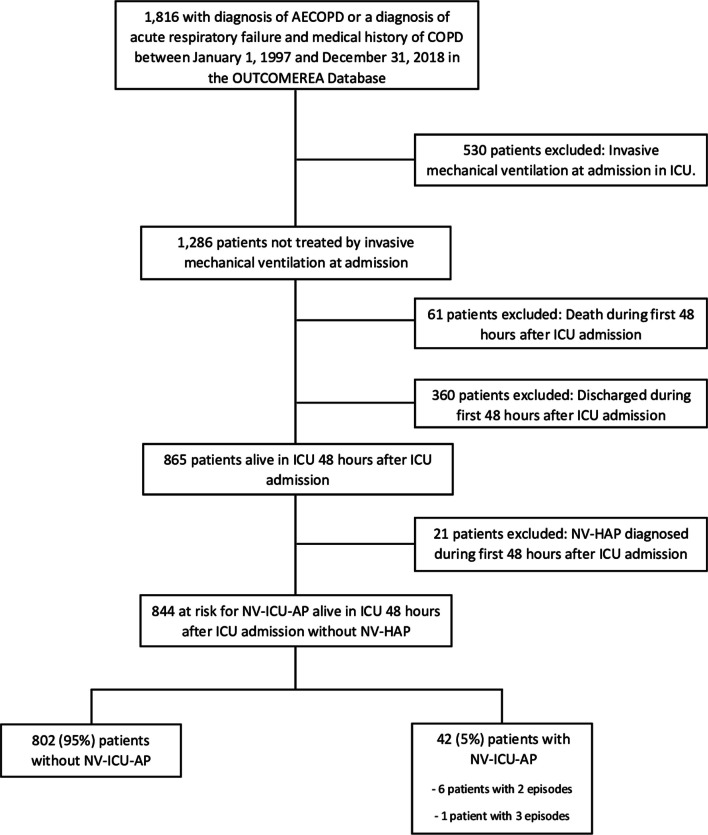
Population Flowchart. *AECOPD* Acute exacerbation of chronic obstructive pulmonary decease; *COPD* chronic obstructive pulmonary disease, *OUTCOMEREA database* multicenter longitudinal database fuelled by ICUs contributing to the OUTCOMEREA Network, *ICU* intensive care unit, *NV-Hospital-AP* non-ventilator hospital-acquired pneumonia, *NV-ICU-AP* non-ventilator-associated intensive care unit acquired pneumonia

All adults admitted to one of the 32 ICUs participating in the prospective national OutcomeRea™ database between 1^st^ January, 1997 and 31^st^ December, 2018, with a diagnosis of *COPD exacerbation* or *acute respiratory failure with a medical history of COPD* were included in the analysis. The exclusion criteria are summarised in Fig. 1.

### Ethics

The French Advisory Committee on Data Processing in Health Research, the French Commission on Informatics and Liberty, and the Ethics Committee of the University of Clermont-Ferrand, France, (IRB No. 5891) approved this data collection (ref. 2007–16). Patients were informed of the inclusion of their de-identified data in the database and could oppose this if they so wished.

### *OutcomeRea*™* database*

OutcomeRea™ is an ongoing, prospective, observational, collaborative, and multicentre database. All codes and definitions used to describe diseases, comorbidities, and outcomes were established before the study began and have been described previously [25]. Clinical and outcome data, treatments, and prescribed medications are prospectively entered into the database daily for a random sample of patients admitted to 32 French ICUs. Of these 32 participating ICUs (including 18 university hospitals), 16 were polyvalent or surgical ICUs and 16 were primarily medical ICUs.

### Methods and measurements

Patient characteristics, prescriptions, and use and type of ventilatory support were extracted from the database. The diagnosis of “very severe COPD” was defined as previous long-term treatment with home oxygen therapy, home NIV, or documented forced expiratory volume in 1 s < 30% predicted value (GOLD classification, stage 4) [1]. Patients who did not meet these criteria, with limited follow-up data, without recent spirometry or without available data were classified as having “not very severe COPD or unknown COPD severity.”

### NV-ICU-AP definition

The risk of NV-ICU-AP was considered from the end of the first 48 h in ICU (without IMV) until ICU discharge, death, or need for at least 48 h of IMV later during their ICU stay. The period considered as risk of VAP ranged from 48 h after intubation to weaning from the invasive ventilation. Pulmonary infection was suspected in patients with a new or persistent infiltrate on the chest radiograph that was associated with any of the following criteria: (1) purulent tracheal secretions, (2) fever ≥ 38.5 °C or hypothermia ≤ 36.5 °C, and (3) leucocytosis > 10^9^ G/L or leucopenia < 4.10^8^ G/L. The diagnosis of NV-ICU-AP was confirmed by bacteriological tests or by a senior investigator in cases of strong clinical suspicion and impossibility of sampling /and or bacteriological tests. The diagnosis of VAP was confirmed by bacteriological tests.

The bacteriological samples considered were a sputum culture (threshold, > 10^5^ cfu/mL of a good-quality sample), bronchoalveolar lavage fluid (threshold, ≥ 10^4^ cfu/mL), plugged telescopic catheter (threshold, ≥ 10^3^ cfu/mL), or a quantitative endotracheal aspirate (threshold, ≥ 10^5^ cfu/mL), received within the first 24 h after suspicion of NV-ICU-AP or VAP. When bacteriological examinations yielded only coagulase-negative *staphylococci* or *Enterococcus* species, a nosocomial pneumonia diagnosis was confirmed only after checking the patient’s data by a senior investigator.

### Definitions

Appropriate antibiotic therapy (AT) required a positive culture result of bacteriological tests, received within the first 24 h after suspicion of NV-ICU-AP. The time to administration of appropriate AT was the number of days before the patient received appropriate AT (in case of inadequate AT or delay to AT prescription) after suspicion of NV-ICU-AP. The presence of multidrug-resistant (MDR) bacteria required documented evidence of at least one of the four classes of MDR: methicillin-resistant Staphylococcus aureus, extended-spectrum β-lactamase–producing Enterobacteriaceae, AmpC-producing Enterobacteriaceae, and Pseudomonas aeruginosa resistant to ticarcillin and/or imipenem and/or ceftazidime in the bacteriological samples.

### Statistical analysis

Categorical parameters are expressed as numbers and percentages, and continuous parameters are given as median and interquartile range [IQR]. For missing data concerning body mass index (BMI), multiple imputations were performed using a fully conditional specification method with linear regression and twenty imputed datasets were created to consider this variable with less than 20% of missing values [26]. Factors associated with the occurrence of NV-ICU-AP were identified using the Fine and Gray competing risk model [27], with 2 days of IMV, discharge from the ICU, and death as competing risks. An adjusted analysis was performed including variables collected at ICU admission, with p < 0.2 in the univariate analysis and considered potentially collinear. The association between NV-ICU-AP and survival on Day-28 was assessed by a Cox model with NV-ICU-AP as a time-dependent variable. To assess the association between NV-ICU-AP and the length of stay in the ICU (using the daily instantaneous risk of alive discharge from the ICU), and association between NV-ICU-AP and intubation, we used a cause-specific regression model controlling for the competing risks of death or of discharge from the ICU with NV-ICU-AP as a time-dependent variable. The association between inadequate antibiotic treatment, time to appropriate antibiotic treatment, and documented presence of MDR bacteria on mortality and intubation, were assessed using the same models. For all the multivariate regression models, variables with p < 0.2 in the univariate models were introduced using a stepwise selection method. In cases of multiple episodes of NV-ICU-AP, only the first episode was considered. All models were stratified by centre, and a 2-sided alpha threshold of 0.05 was considered statistically significant. Statistical analyses were performed using SAS 9.4 software (SAS Institute, Cary, NC, USA).

## Results

### Study flow

**Fig. 2:**
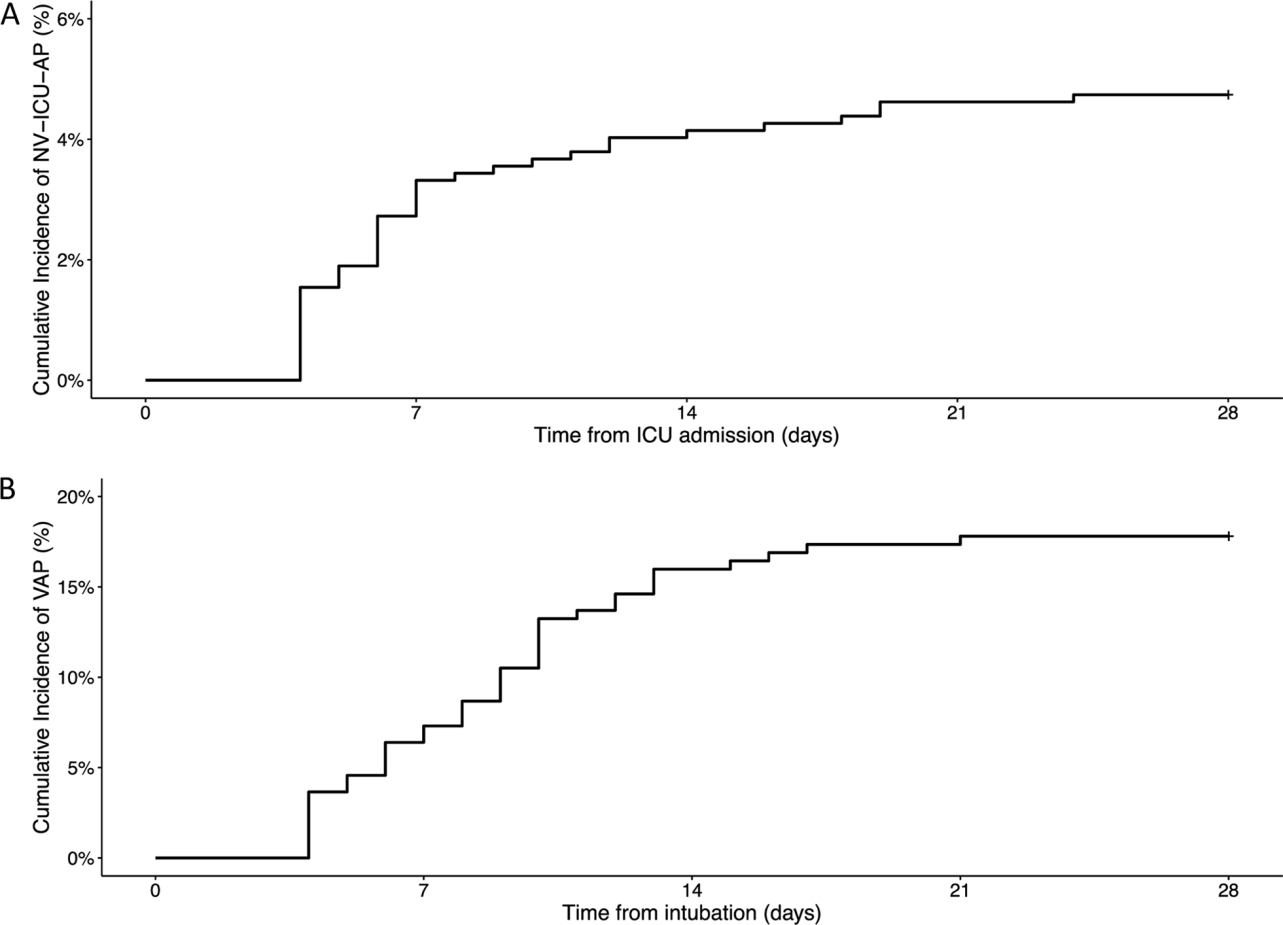
Cumulative incidences of non-ventilator-associated ICU-acquired pneumonia and ventilator-associated pneumonia in patients admitted in ICU for a severe acute exacerbation of COPD. **A** Cumulative incidence of non-ventilator-associated ICU-acquired pneumonia from ICU admission in patients admitted to an ICU for a severe acute exacerbation of COPD. **B** Cumulative incidence of ventilator-associated pneumonia from intubation in ICU for a severe acute exacerbation of COPD (n = 219). *ICU* intensive care unit, *NV-ICU-AP* non-ventilator-associated intensive care unit acquired pneumonia, *VAP* ventilator-associated pneumonia

Among the 23,249 patients registered in the OutcomeRea™ database between 1^st^ January 1997 and 31st December 2018, 1,816 patients had a diagnosis of *acute exacerbation of COPD* or *acute respiratory failure with a medical history of COPD*. Of these 844 were not initially given IMV and were thus were exposed to the risk of NV-ICU-AP. Fifty episodes of NV-ICU-APs occurred in 42 patients (six patients presented two episodes of NV-ICU-AP, and one patient had 3 episodes) with an incidence density of 10.8 per 1,000 patients-days exposed to the risk of NV-ICU-AP (Figs. 1 and 2). The first episode of NV-ICU-AP occurred after a median delay of 6 [4; 11] days after ICU admission, and 32 patients with NV-ICU-AP (76.2%) were intubated within 48 h after diagnosis of NV-ICU-AP (Table 1). The baseline characteristics and outcomes of patients are presented in Table 2. The main microorganisms found were *S. aureus*, *P. aeruginosa*, and *Escherichia Coli*. Six (14.29%) NV-ICU-AP cases were diagnosed without bacteriological documentation (Table 1). MDR bacteria were found in 13 of 36 patients (36%) with a first episode of NV-ICU-AP (with bacteriological documentation) and mainly concerned *S. aureus* and *P. aeruginosa* (Table 1). 219 patients were intubated and 59 episodes of VAP occurred in 39 patients with an incidence density of 23.1 per 1,000 patients-days at risk of VAP (Fig. 2). The first episode of VAP occurred after a median delay of 11 [8; 14] days after ICU admission and after 9 [5; 11] days of invasive mechanical ventilation. Baseline characteristics and outcomes of patients according to the occurrence of VAP are shown in Additional file 1: Tables S1 and S2.

**Table 1:** Baseline characteristics and mortality rate for patients with non-ventilator-associated ICU-acquired pneumonia admitted to an ICU for severe acute exacerbation of chronic obstructive pulmonary disease

	No NV-ICU-AP (n = 802)	NV-ICU-AP (n = 42)	p Value
	Median [Q1; Q3] or n (percentage)	Median [Q1; Q3] or n (percentage)	
**Baseline characteristics**			
Age (years)	70.7 [62.0; 78.1]	72.3 [67.6; 76.9]	0.33
Male sex, n (%)	499 (62.2)	31 (73.8)	0.13
BMI (kg/m^2^)	24.9 [20.8; 30.3]	23.9 [21.2; 30.1]	0.74
SAPS II score	34.0 [26.0; 42.0]	38.0 [30.0; 45.0]	0.05
Maximum SOFA Day 1–Day 2	4.0 [3.0; 6.0]	5.0 [3.0; 6.0]	0.06
Hospitalisation before ICU admission (yes), n (%)	264 (32.9)	20 (47.6)	0.05
Immunodeficiency (yes), n (%)	74 (9.2)	6 (14.3)	0.28
No decrease in consciousness Day 1–Day 2 (Glasgow Coma Scale = 15)	490 (61.1)	14 (33.1)	< 0.01
MDR bacterial colonisation, (yes), n (%)	47 (5.9)	3 (7.1)	0.73
**COPD severity**			
Very severe COPD, n (%)	173 (21.6)	1 (2.4)	< 0.01
**Trigger of the acute exacerbation of COPD**			
Respiratory infection, n (%)	524 (65.3)	29 (69.0)	0.74
Non-infectious respiratory causes, n (%)	165 (20.6)	8 (19.0)	
Cardiac and thromboembolic events, n (%)	63 (7.9)	4 (9.5)	
Others, n (%)	50 (6.2)	1 (2.4)	
**Therapeutic limitation**			
Limitation of therapeutic effort at admission to ICU, (yes) n (%)	63 (7.9)	3 (7.1)	0.87
**Corticosteroid therapy**			
Use of corticosteroids therapy at admission, (yes) n (%)	302 (37.7)	12 (28.6)	0.24
**Antibiotic therapy**			
Use of antibiotic therapy at admission, (yes) n (%)	561 (70.0)	25 (59.5)	0.15
**Gastroprotective agents**			
Use of gastroprotective agents at admission, (yes) n (%)	411 (51.2)	22 (52.4)	0.89
**Enteral nutrition**			
Use of enteral nutrition at admission, (yes) n (%)	99 (12.3)	10 (23.8)	0.03
**Lengths of stay**			
ICU Length of stay (days)	6.0 [5.0; 10.0]	24.5 [14.0; 37.0]	< 0.01
Hospital Length of stay (days)	18.0 [12.0; 30.0]	37.0 [22.0; 59.0]	< 0.01
**Mortality**			
ICU Mortality rate, n (%)	73 (9.1)	16 (38.1)	< 0.01
Hospital Mortality rate, n (%)	123 (15.3)	18 (42.9)	< 0.01
Mortality at Day 28, n (%)	96 (12.0)	10 (23.8)	0.02
**Non-ventilator-associated ICU-acquired pneumonia**			
Day of first diagnosis of NV-ICU-AP (days in ICU)	–	6.0 [4.0; 11.0]	
Day of first diagnosis of NV-ICU-AP (days in hospital)	–	7.5 [5.0; 16.0]	
NV-ICU-AP requiring intubation, (yes) n (%)	–	32 (76.2)	

Very Severe COPD = Oxygen therapy at home or NIV at home or airflow limitation Stage 4. The use of corticosteroids therapy at admission was defined as a daily dose ≥ 0.5 mg/kg of prednisone or equivalent prescribed during the first 24 h after admission in ICU for the current AECOPD. Immunodeficiency was defined by the presence of aplasia, corticosteroid therapy for more than one month or at a dose > 2 mg/kg of prednisone equivalent, chemotherapy, and human immunodeficiency virus (HIV) at the acquired immunodeficiency syndrome (AIDS) stage or organ transplantation. Bacterial colonisation was defined by the presence of MDROs on screening samples taken on admission in ICU. These MDROs correspond to methicillin-resistant Staphylococcus aureus, extended-spectrum β-lactamase–producing Enterobacteriaceae, AmpC-producing Enterobacteriaceae, and Pseudomonas aeruginosa resistant to ticarcillin and/or imipenem and/or ceftazidime in the bacteriological samples

*ICU* Intensive Care Unit, *BMI* Body Mass Index, *SAPS II* Simplified Acute Physiology Score II, *SOFA Score* Sequential Organ Failure Assessment Score, *COPD* Chronic Obstructive Pulmonary Disease, *NV-ICU-AP* Non-ventilator-associated Intensive Care Unit Acquired Pneumonia

**Table 2:** Microorganisms causing non-ventilator-associated ICU-acquired pneumonia in patients admitted to an ICU for severe acute exacerbation of chronic obstructive pulmonary disease

Microorganism	n (%)
**Gram-positive bacteria**	
***Staphylococcus aureus***	8 (19.05)
Methicillin susceptible	6 (14.29)
Methicillin resistant	2 (4.76)
**Other Gram-positive microorganisms**	4 (9.52)
**Gram-negative bacteria**	
***Escherichia coli***	7 (16.67)
Susceptible to third-generation cephalosporin	6 (14.29)
Resistant to third-generation cephalosporin	1 (2.38)
***Pseudomonas aeruginosa***	11 (26.19)
Susceptible	9 (21.43)
Resistant to ticarcillin, ceftazidime, or penems	2 (4.76)
*Haemophilus influenzae*	4 (9.52)
*Stenotrophomonas maltophilia*	4 (9.52)
*Citrobacter freundii*	1 (2.38)
*Enterobacter aerogenes*	3 (7.14)
*Enterobacter cloacae*	2 (4.76)
*Morganella morganii*	1 (2.38)
*Serratia marcescens*	2 (4.76)
** Other Gram-negative microorganisms**	1 (2.38)
**Cases without bacteriological documentation**	6 (14.29)
**More than one microorganism**	11 (23.91)

*ICU* intensive care unit

We give the bacteriological results for the first episode of NV-ICU-AP during the patient’s stay in intensive care, i.e. 42 occurrences of NV-ICU-APs

#### Factors associated with NV-ICU-AP occurrence

In the adjusted analysis, the prescription of empirical antibiotic therapy at ICU admission (Sub-Distribution Hazard Ratio (sdHR) = 0.45 [0.23; 0.86], p = 0.02) and no decrease in consciousness (Glasgow Coma Scale = 15) during the two first days in the ICU (sdHR = 0.35 [0.16; 0.76], p < 0.01) were associated with a lower risk of NV-ICU-AP. Age, sex, severity of COPD, corticosteroid therapy, or non-invasive ventilation (NIV) at admission were not associated with an increased risk of NV-ICU-AP (Additional file 1: Table S3). Data and outcomes by the level of consciousness are summarised in the Additional file 1: Table S4.

#### NV-ICU-AP and mortality, intubation, and length of stay in ICU.

At Day 28, 40 patients had undergone at least one episode of NV-ICU-AP (two patients had NV-ICU-AP after D28). For the survival analysis, we excluded 66 patients with limitation of therapeutic effort (LTE) at admission to the ICU, among whom only three developed NV-ICU-AP at before D28. NV-ICU-AP was an independent risk factor for mortality after adjustment for other risk factors for in-hospital death (Hazard Ratio (HR) = 3.03 [1.36; 6.73], p < 0.01) (Additional file 1: Table S5). The negative effect of NV-ICU-AP on mortality was even greater if the patient was intubated shortly after the diagnosis of NV-ICU-AP (for 48 h maximum) (HR = 4.23 [1.88; 9.55], p < 0.01).

The occurrence of NV-ICU-AP was strongly associated with an increased risk of intubation (Cause-specific hazard ratios (csHR) = 4.98 [2.53; 9.80], p < 0.01) for 48 h maximum after the diagnosis of NV-ICU-AP (Additional file 1: Tables S6 and S7), and with an increase in the length of stay in the ICU of 9.8 [8.2; 16.3] days (p < 0.01) (Additional file 1: Tables S8 and S9).

#### Antibiotic treatment

Among the 36 patients with a microbiologically documented first occurrence of NV-ICU-AP, 20 patients received appropriate antibiotic therapy within the first 24 h after developing NV-ICU-AP. For the 16 patients with a delay in receiving appropriate AT, the median delay was 2 [1.0; 3.0] days. After adjustment, inadequate AT, time to appropriate AT, and the implication of an MDR bacterium in the pneumonia was not associated with survival or intubation (Additional file 1: Tables S10 to S14).

## Discussion

To our knowledge, this is the first study addressing the impact of NV-ICU-AP on the outcomes of patients with COPD admitted to an ICU for AECOPD. Prescription of empirical antibiotic therapy at ICU admission and a good level of consciousness were associated with a lower risk of NV-ICU-AP. In this specific population, NV-ICU-AP is an independent risk factor for 28-day mortality, intubation, and increased ICU length of stay. The early prescription of antibiotics, close to ICU admission, and a good level of consciousness were associated with a lower risk of NV-ICU-AP.

In our observational study, the incidence density (10.8 per 1,000 patient-days) and incidence (5%) of NV-ICU-AP are higher than those reported in the literature (0.6 to 4.5 per 1000 patient-days and 1.6–2.5%, respectively [5, 8, 13, 14]) in unselected ICU populations. They are closer to the incidence of nosocomial pneumonia of 3.1% (corresponding to 4.5 per 1000 NIV-days) in ICU patients treated by NIV [23]. This higher incidence of NV-ICU-AP in COPD patients is consistent with the results of studies of NV-HAP outside of ICUs, where chronic bronchitis/emphysema was a risk factor for NV-HAP [8, 18–21].

Other risk factors for NV-HAP identified in previous studies were male sex, older age, comorbidities, immunosuppression [6, 8, 17, 19], and ICU admission [17, 19]. In our population, we found the risk of NV-ICU-AP decreased with better levels of consciousness (as rated by the Glasgow score). Diminished consciousness has already been identified as a risk factor for HAP outside of ICUs, owing to a reduced ability to protect the airways, thus increasing the risk of aspiration of pathogens [8, 28, 29]. NV-ICU-AP was less frequent in patients who started a course of antibiotics (empirical then appropriate) upon ICU admission. However, our data alone are not sufficient to promote systematic antibiotics, especially until the effect of this antibiotic therapy on the acquisition of MDROs in this setting has not been evaluated. The patients with very severe COPD showed a trend towards to a protective effect on the occurrence of NV-ICU-AP. Therefore, the ICU practitioners need to carefully monitor the occurrence of NV-ICU-AP, also in patients with less severe COPD. However, an important limitation of this result is the presence of a group of patients with unknown COPD severity. In fact, some patients had limited follow-up data or no recent spirometry.

The hospital mortality of patients with NV-ICU-AP reported in the literature is 22 to 36%, increasing to 48% when NV-ICU-AP results in intubation, and is significantly higher than the mortality of patients without NV-ICU-AP [5, 13, 14, 22]. The in-hospital mortality of patients in our population who developed NV-ICU-AP was 43%, higher than in previous studies of unselected ICU populations. Nevertheless, it remained lower than the in-hospital mortality rate of 75% reported by *Zhang *et al*.* [23] in patients with NV-ICU-PA treated with NIV; most of whom suffered from COPD. We found the occurrence of NV-ICU-AP to be independently associated with an increased risk of death up to D 28 (HR = 3.03 [1.36; 6.73]), which was higher than in the general ICU population (HR 30-day mortality = 1.82 [1.35; 2.45] according to *Saied *et al. [14]). We found an even higher risk in cases requiring intubation after the diagnosis of NV-ICU-AP (HR = 4.23 [1.88; 9.55]), and *Vallecoccia *et al*.* reported that HAs requiring mechanical ventilation had the greatest 28-day mortality rate among all types of nosocomial pneumonia, including VAPs [9]. Therefore, the need for intubation should alert the ICU teams to the high severity and the poor prognosis of the patients with NV-ICU-AP.

We found that 76% of patients with NV-ICU-AP were intubated shortly after NV-ICU-AP diagnosis and that NV-ICU-AP was associated with an independent risk of intubation (csHR, 4.98 [2.53; 9.80]), similar to the intubation rate (63 to 75%) [16, 23] and risk of intubation in patients in the ICU on NIV or oxygen therapy (OR: 6.74; 95% CI: 2.24–20.28) [23, 30].

Given these high rates of intubation and mortality, we investigated the possibility that delayed intubation was responsible for these poor outcomes. Only 4 patients had criteria for the use of invasive mechanical ventilation the day before intubation, and they were alive at ICU discharge (Additional file 1: Table S15). Therefore, a possible delay in intubation cannot explain the poor outcome of the patients in our study.

In accordance with the recommendations of the Surviving Sepsis Campaign [31] and the European treatment guidelines for HAPs and VAPs [3], patients with pneumonia suspected as being due to bacterial infection should be given empirical antibiotic treatment as early as possible, and switched to the appropriate antibiotic once the results of bacteriological tests are known. We found no association between outcomes and inappropriate antibiotic therapy in patients with NV-ICU-AP. This finding should be interpreted with great caution given the small number of events in our study. Several observational studies and a meta-analysis have examined the effect of inappropriate initial antibiotic therapy in cases of nosocomial pneumonia in the ICU, mainly VAP. Inappropriate initial antibiotic therapy has been associated with worse outcomes in terms of duration of mechanical ventilation, lengths of stay, and mortality [32–35].

To summarise the specificities of patients admitted to an ICU with AECOPD, (compared to the few studies of NV-ICU-AP among the general ICU population [5, 8, 14, 16]), we note the higher incidence of NV-ICU-AP with a strong association with survival, intubation, and long length of ICU stay. The high incidence of NV-ICU-AP among patients admitted with AECOPD was not explained by the use of NIV on ICU admission (Additional file 1: Table S3), although it has been suggested that NIV could promote the risk of pneumonia due to oesophageal or stomach distension [36]. COPD has previously been described as a risk factor for HAP outside of ICUs [18] and this could be explained by mucus production in patients with chronic bronchitis, the presence of pathogenic bacteria in the airways, increased inflammation, and the host’s immune response. These hypotheses might also be proposed for ICU patients.

Consistent with our results, *Zhang *et al*.* [23] and *Rinaudo *et al*.* [22] reported that 90-day mortality rates of patients with vs. without COPD who developed NV-ICU-AP tended to be significantly to be different (22/41 [54%] vs. 29/82 [36%], respectively; p = 0.06); COPD patients seem to be a population at risk of poor outcomes in case of NV-ICU-AP.

Patients in our study were frequently intubated in the event of NV-ICU-AP, had a long duration of ICU stay (25 days) and 75% mortality. The length of stay compares to 14–20 days for *Esperatti *et al*.* [5] and *Saied *et al*.* [14], 11 days for *Zhang *et al*.* [23]. This long duration of ICU stay in our population of COPD patients could be explained by the fact that COPD is associated with an increase in the length of ICU stay and the duration of invasive ventilation [37], with a longer weaning period from invasive ventilation [38].

Strengths of our study are that we used the well-established high-quality OutcomeRea™ database, and statistical analyses were controlled for competing risks. There were several limitations. It was possible for clinicians to declare NV-ICU-AP without a bacteriological diagnosis (recorded in the database) in cases of strong clinical suspicion and impossibility of bacteriological sampling. Unsurprisingly, antibiotic therapy at ICU admission was a protective factor against the occurrence of NV-ICU-AP, but we did not have data on previous antibiotic treatments received at home or outside the ICU during the weeks preceding ICU admission. Such data could help us understand the bacterial environment of patients and influence the risk of NV-ICU-AP, as has been previously demonstrated [16], and may be crucial when multidrug-resistant organisms (MDRO) are implicated [9]. The European guidelines for nosocomial pneumonia [3] include the following risk factors for MDRO infection: a hospital environment with high levels of MDROs, prior use of antibiotics, recent hospitalisation (> 5 days), and prior infection by an MDRO.

A major concern at ICU admission must be the better identification of patients at high risk of developing NV-ICU-AP, as patients with COPD seem to be. Clinical trials are needed to determine the optimal preventive strategies, diagnostic tools (including fast reliable molecular diagnostic techniques) and ways to improve the management of NV-ICU-AP (including new therapeutic agents or strategies) [8, 9, 16, 39]. Indeed, insufficient knowledge of NV-ICU-AP and insufficient codified diagnostic methods in these non-ventilated patients may lead to underdiagnosis, diagnostic delay, and poor prognosis [7, 40].

## Conclusion

The occurrence of NV-ICU-AP was high in this large population of patients admitted to French ICUs with severe AECOPD. Prescription of empirical antibiotic therapy on ICU admission and a good level of consciousness were associated with a lower risk of NV-ICU-AP. NV-ICU-AP was associated with an increased risk of death before day 28, intubation, and prolonged ICU stay. The prognosis of these patients could be improved by the implementation of preventive measures and greater efficiency in the diagnosis of NV-ICU-AP.

### Supplementary Information

**Additional file 1**. Members of the OutcomeRea Network.

## Data Availability

The datasets used and/or analysed during the current study are available from the corresponding author on reasonable request.

## Abbreviations

COPD: Chronic obstructive pulmonary disease
AECOPD: Acute exacerbation of chronic obstructive pulmonary disease
ICU: Intensive care unit
VAP: Ventilator-associated pneumonia
IMV: Invasive mechanical ventilation
NV-ICU-AP: Non-ventilator-associated ICU-acquired pneumonia
NV-HAP: Non-ventilator hospital-acquired pneumonia
HAP: Hospital-acquired pneumonia
NIV: Non-invasive ventilation
GOLD: Global Initiative for Chronic Obstructive Lung Disease
Cfu/mL: Colony-forming unit/millilitre
AT: Antibiotic therapy
MDR: Multidrug-resistant
IQR: Interquartile range
BMI: Body mass index
sdHR: Sub-distribution hazard ratio
HR: Hazard Ratio
csHR: Cause-specific hazard ratio
OR: Odds ratio
MDRO: Multidrug-resistant organisms
