# Correction: Non-ventilator-associated ICU-acquired pneumonia (NV-ICU-AP) in patients with acute exacerbation of COPD: From the French OUTCOMEREA cohort

**DOI:** 10.1186/s13054-024-04864-9

**Published:** 2024-04-09

**Authors:** Louis‑Marie Galerneau, Sebastien Bailly, Nicolas Terzi, Stephane Ruckly, Maite Garrouste‑Orgeas, Johanna Oziel, Vivien Hong Tuan Ha, Marc Gainnier, Shidasp Siami, Claire Dupuis, Jean‑Marie Forel, Anais Dartevel, Julien Dessajan, Christophe Adrie, Dany Goldgran‑Toledano, Virginie Laurent, Laurent Argaud, Jean Reignier, Jean‑Louis Pepin, Michael Darmon, Jean‑Francois Timsit, OUTCOME R. E. A. network

**Affiliations:** 1 Medical Intensive Care Unit, University Hospital of Grenoble Alpes, 10217 38043 Grenoble, CS France; 2 Grenoble Alpes University, INSERM 1300, HP2, Grenoble, France; 3 Department of Biostatistics, Outcomerea, Paris, France; 4 Medical Unit, French and British Hospital Cognacq-Jay Fondation, Levallois‑Perret, France; 5 Intensive Care Unit, Avicenne Hospital, AP-HP, Paris, France; 6 Medical Intensive Care Unit, Meaux Hospital, Meaux, France; 7 Medical Intensive Care Unit, La Timone Hospital, Marseille, France; 8 Critical Care Medicine Unit, Etampes-Dourdan Hospital, Etampes, France; 9 Medical Intensive Care Unit, Gabriel Montpied University Hospital, Clermont‑Ferrand, France; 10 Medical Intensive Care Unit, Nord University Hospital, Marseille, France; 11 Medical and Infectious Diseases Intensive Care Unit (MI2), Bichat Hospital, AP-HP, Paris, France; 12 Polyvalent Intensive Care Unit, Delafontaine Hospital, Saint‑Denis, France; 13 Medical Intensive Care Unit, Le Raincy-Montfermeil Hospital, Montfermeil, France; 14 Intensive Care Unit, Andre Mignot Hospital, Le Chesnay, France; 15 Medical Intensive Care Unit, Edouard Herriot Hospital, Lyon Civil Hospices, Lyon, France; 16 Medical Intensive Care Unit, Nantes University Hospital, Nantes, France; 17 Intensive Care Unit, Saint-Louis Hospital, AP-HP, Paris, France

**Correction:Crit Care (2023) 27:359** 10.1186/s13054-023-04631-2

Following publication of the original article [1], the authors identified an error in Table 1. The results were inverted between for variable No decrease in consciousness Day 1–Day 2. The correct table is given hereafter.

**Table 1:** Baseline characteristics and mortality rate for patients with non-ventilator-associated ICU-acquired pneumonia admitted to an ICU for severe acute exacerbation of chronic obstructive pulmonary disease

	No NV-ICU-AP (n = 802)	NV-ICU-AP (n = 42)	*p* value
	Median [Q1; Q3] or n (percentage)	Median [Q1; Q3] or n (percentage)	
**Baseline characteristics**			
Age (years)	70.7 [62.0; 78.1]	72.3 [67.6; 76.9]	0.33
Male sex, n (%)	499 (62.2)	31 (73.8)	0.13
BMI (kg/m^2^)	24.9 [20.8; 30.3]	23.9 [21.2; 30.1]	0.74
SAPS II score	34.0 [26.0; 42.0]	38.0 [30.0; 45.0]	0.05
Maximum SOFA Day 1- Day 2	4.0 [3.0; 6.0]	5.0 [3.0; 6.0]	0.06
Hospitalisation before ICU admission (yes), n (%)	264 (32.9)	20 (47.6)	0.05
Immunodeficiency (yes), n (%)	74 (9.2)	6 (14.3)	0.28
No decrease in consciousness Day 1- Day 2 (Glasgow Coma Scale = 15)	490 (61.1)	14 (33.1)	< 0.01
MDR bacterial colonization, (yes), n (%)	47 (5.9)	3 (7.1)	0.73
**COPD severity**			
Very Severe COPD, n (%)	173 (21.6)	1 (2.4)	< 0.01
**Trigger of the acute exacerbation of COPD**			
Respiratory infection, n (%)	524 (65.3)	29 (69.0)	0.74
Non-infectious respiratory causes, n (%)	165 (20.6)	8 (19.0)	
Cardiac and thromboembolic events, n (%)	63 (7.9)	4 (9.5)	
Others, n (%)	50 (6.2)	1 (2.4)	
**Therapeutic limitation**			
Limitation of therapeutic effort at admission to ICU, (yes) n (%)	63 (7.9)	3 (7.1)	0.87
**Corticosteroid therapy**			
Use of corticosteroids therapy at admission, (yes) n (%)	302 (37.7)	12 (28.6)	0.24
**Antibiotic therapy**			
Use of antibiotic therapy at admission, (yes) n (%)	561 (70.0)	25 (59.5)	0.15
**Gastroprotective agents**			
Use of gastroprotective agents at admission, (yes) n (%)	411 (51.2)	22 (52.4)	0.89
**Enteral nutrition**			
Use of enteral nutrition at admission, (yes) n (%)	99 (12.3)	10 (23.8)	0.03
**Lengths of stay**			
ICU Length of stay (days)	6.0 [5.0; 10.0]	24.5 [14.0; 37.0]	< 0.01
Hospital Length of stay (days)	18.0 [12.0; 30.0]	37.0 [22.0; 59.0]	< 0.01
**Mortality**			
ICU Mortality rate, n (%)	73 (9.1)	16 (38.1)	< 0.01
Hospital Mortality rate, n (%)	123 (15.3)	18 (42.9)	< 0.01
Mortality at Day 28, n (%)	96 (12.0)	10 (23.8)	0.02
**Non-ventilator-associated ICU-acquired pneumonia**			
Day of first diagnosis of NV-ICU-AP (days in ICU)	–	6.0 [4.0; 11.0]	
Day of first diagnosis of NV-ICU-AP (days in hospital)	–	7.5 [5.0; 16.0]	
NV-ICU-AP requiring intubation, (yes) n (%)	–	32 (76.2)	

Very Severe COPD = Oxygen therapy at home or NIV at home or Airflow limitation Stage 4. The use of corticosteroids therapy at admission was defined as a daily dose ≥ 0.5 mg/kg of prednisone or equivalent prescribed during the first 24 h after admission in ICU for the current AECOPD. Immunodeficiency was defined by the presence of aplasia, corticosteroid therapy for more than one month or at a dose > 2mg/kg of prednisone equivalent, chemotherapy, human immunodeficiency virus (HIV) at the acquired immunodeficiency syndrome (AIDS) stage or organ transplantation. Bacterial colonisation was defined by the presence of MDROs on screening samples taken on admission in ICU. These MDROs correspond to methicillin-resistant Staphylococcus aureus, extended-spectrum β-lactamase–producing Enterobacteriaceae, AmpC-producing Enterobacteriaceae, and Pseudomonas aeruginosa resistant to ticarcillin and/or imipenem and/or ceftazidime in the bacteriological samples

ICU, Intensive Care Unit; BMI, Body Mass Index, SAPS II, Simplified Acute Physiology Score II, SOFA Score, Sequential Organ Failure Assessment Score; COPD, Chronic Obstructive Pulmonary Disease, NV-ICU-AP, Non-ventilator-associated Intensive Care Unit Acquired Pneumonia

The incorrect Table 1 values are:

In the No NV-ICU-AP population, the number of patients with No decrease in consciousness Day 1–Day 2 (Glasgow Coma Scale = 15) is 312 (38.9%)

In the NV-ICU-AP population, the number of patients with No decrease in consciousness Day 1–Day 2 (Glasgow Coma Scale = 15) is 28 (66.7%)

The correct Table 1 values are:

In the No NV-ICU-AP population, the number of patients with No decrease in consciousness Day 1–Day 2 (Glasgow Coma Scale = 15) is 490 (61.1%)

In the NV-ICU-AP population, the number of patients with No decrease in consciousness Day 1–Day 2 (Glasgow Coma Scale = 15) is 14 (33.1%)

Table 1 has been updated in this correction article and the original article [1] has been corrected.
