# Supplementary material for: Non-ventilator-associated ICU-acquired pneumonia (NV-ICU-AP) in patients with acute exacerbation of COPD: From the French OUTCOMEREA cohort
Source: Crit Care. 2023 Sep 19;27:359. doi: 10.1186/s13054-023-04631-2 (PMC10508006; doi:10.1186/s13054-023-04631-2)
Supplement: Supplementary file 1 — Additional file 1. Members of the OutcomeRea Network. [file 13054_2023_4631_MOESM1_ESM.docx]

**Non-ventilator-associated ICU-acquired pneumonia (NV-ICU-AP) in patients with acute exacerbation of COPD: From the French OUTCOMEREA cohort**

**Supplemental Table 1. Baseline characteristics and outcomes for patients with and without ventilator-associated pneumonia admitted in ICU for severe acute exacerbation of chronic obstructive pulmonary disease.**

|  | No IMV (n=625) | IMV without VAP (n=180) | IMV with VAP (n=39) | p Value |
| --- | --- | --- | --- | --- |
|  | Median [Q1; Q3] or n (percentage) | Median [Q1; Q3] or n (percentage) | Median [Q1; Q3] or n (percentage) |  |
| **Baseline characteristics** | | | | |
| Age (years) | 70.8 [62.7; 78.2] | 70.5 [61.9; 77.4] | 71.5 [65.8; 77.1] | 0.77 |
| Male sex, n (%) | 384 (61.4) | 115 (63.9) | 31 (79.5) | 0.07 |
| BMI (kg/m^2^) | 24.9 [20.9; 30.5] | 24.9 [20.2; 30.2] | 25.9 [21.5; 29.4] | 0.92 |
| SAPS II score | 33.0 [25.0; 40.0] | 38.0 [30.0; 49.0] | 37.0 [30.0; 44.0] | <0.01 |
| Maximum SOFA Day 1- Day 2 | 4.0 [2.0; 5.0] | 5.0 [4.0; 7.0] | 6.0 [3.0; 7.0] | <0.01 |
| Hospitalisation before ICU admission (yes), n (%) | 201 (32.2) | 70 (38.9) | 13 (33.3) | 0.24 |
| Immunodeficiency (yes), n (%) | 45 (7.2) | 26 (14.4) | 9 (23.1) | <0.01 |
| No decrease in consciousness Day 1- Day 2 (Glasgow Coma Scale = 15) | 447 (71.5) | 48 (26.7) | 9 (23.1) | <0.01 |
| MDR bacterial colonisation, (yes), n (%) | 33 (5.3) | 14 (7.8) | 3 (7.7) | 0.41 |
| **COPD Severity** | | | | |
| Very Severe COPD, n (%) | 144 (23.0) | 27 (15.0) | 3 (7.7) | <0.01 |
| **Trigger of the acute exacerbation of COPD** | | | | |
| Respiratory infection, n (%) | 410 (65.5) | 122 (67.8) | 21 (53.8) | 0.6 |
| Non-infectious respiratory causes, n (%) | 129 (20.6) | 34 (18.9) | 10 (25.6) |  |
| Cardiac and thromboembolic events, n (%) | 51 (8.2) | 11 (6.1) | 5 (12.8) |  |
| Others, n (%) | 35 (5.6) | 13 (7.2) | 3 (7.7) |  |
| **Therapeutic limitation** | | | | |
| Limitation of therapeutic effort at admission to ICU, (yes) n (%) | 50 (8.0) | 13 (7.2) | 3 (7.7) | 0.94 |
| **Corticosteroid Therapy** | | | | |
| Use of corticosteroids therapy at admission, (yes) n (%) | 223 (35.7) | 70 (38.9) | 21 (53.8) | 0.06 |
| **Antibiotic Therapy** | | | | |
| Use of antibiotic therapy at admission, (yes) n (%) | 424 (67.8) | 135 (75.0) | 27 (69.2) | 0.18 |
| **Gastroprotective agents** | | | | |
| Use of gastroprotective agents at admission, (yes) n (%) | 312 (49.9) | 100 (55.6) | 21 (53.8) | 0.39 |
| **Enteral Nutrition** | | | | |
| Use of enteral nutrition at admission, (yes) n (%) | 12 (1.9) | 80 (44.4) | 17 (43.6) | <0.01 |
| **Lengths of stay** | | | | |
| ICU Length of stay (days) | 6.0 [5.0; 8.0] | 14.0 [8.5; 22.0] | 27.0 [21.0; 41.0] | <0.01 |
| Hospital Length of stay (days) | 17.0 [11.0; 27.0] | 26.0 [16.0; 44.0] | 41.0 [26.0; 58.0] | <0.01 |
| **Mortality** | | | | |
| ICU Mortality rate, n (%) | 27 (4.3) | 46 (25.6) | 16 (41.0) | <0.01 |
| Hospital Mortality rate, n (%) | 63 (10.1) | 61 (33.9) | 17 (43.6) | <0.01 |
| Mortality at Day 28, n (%) | 53 (8.5) | 43 (23.9) | 10 (25.6) | <0.01 |
| **Invasive mechanical ventilation** | | | | |
| Day of intubation (days in ICU) |  | 3.0 [2.0; 4.0] | 2.0 [2.0; 3.0] | 0.37 |
| Intubation after NIV failure |  | 128 (71.1) | 19 (48.7) | <0.01 |
| Length of invasive mechanical ventilation (days) |  | 8.0 [4.0; 14.0] | 23.0 [13.0; 36.0] | <0.01 |

***Abbreviations:*** *ICU = Intensive Care Unit; BMI = Body Mass Index; SAPS II = Simplified Acute Physiology Score II; SOFA Score = Sequential Organ Failure Assessment Score; MDR = Multidrug-resistant; COPD = Chronic Obstructive Pulmonary Disease; NIV = Non-Invasive Ventilation*

***Notes:*** *Very Severe COPD = Oxygen therapy at home or NIV at home or Airflow limitation Stage 4. The use of corticosteroids therapy at admission was defined as a daily dose ≥ 0.5 mg/kg of prednisone or equivalent prescribed during the first 24 hours after admission in ICU for the current AECOPD. The immunodeficiency was defined by the presence of aplasia, corticosteroid therapy lasting more than one month or with a dosage > 2mg/kg of prednisone equivalent, chemotherapy, human immunodeficiency virus (HIV) at the acquired immunodeficiency syndrome (AIDS) stage or organ transplantation. Bacterial colonisation was defined by the presence MDRO on the screening samples performed at admission in ICU. These MDROs correspond to methicillin-resistant Staphylococcus aureus, extended-spectrum β-lactamase–producing Enterobacteriaceae, AmpC-producing Enterobacteriaceae, and Pseudomonas aeruginosa resistant to ticarcillin and/or imipenem and/or ceftazidime in the bacteriological samples.*

**Supplemental Table 2. Microorganisms causing ventilator-associated pneumonia in patients admitted in ICU for severe acute exacerbation of chronic obstructive pulmonary disease.**

| **Microorganism** | **VAP, n (%)** |
| --- | --- |
| **Gram Positive bacteria** | |
| ***Staphylococcus aureus*** | 8 (13.56) |
| Methicillin susceptible | 3 (5.08) |
| Methicillin resistant | 5 (8.47) |
| **Other Gram-positive microorganisms** | 4 (6.78) |
| **Gram negative bacteria** | |
| ***Escherichia coli*** | 7 (11;86) |
| Susceptible to third generation cephalosporin | 5 (8.47) |
| Resistant to third generation cephalosporin | 2 (3.39) |
| ***Pseudomonas aeruginosa*** | 30 (50.85) |
| Susceptible | 22 (37.29) |
| Resistant to ticarcillin, ceftazidime or penems | 8 (13.56) |
| ***Haemophilus influenzae*** | 2 (3.39) |
| ***Stenotrophomonas maltophilia*** | 5 (8.47) |
| ***Citrobacter freundii*** | 2 (3.39) |
| ***Enterobacter cloacae*** | 4 (6.78) |
| ***Morganella morganii*** | 1 (1.69) |
| ***Serratia marcescens*** | 2 (3.39) |
| ***Klebsiella*** | 1 (1.69) |
| ***Proteus Mirabilis*** | 2 (3.39) |
| **Other Gram-negative microorganisms** | 3 (5.08) |
|  | |
| **More than one microorganism** | 11 (18.64) |

***Abbreviations:*** *ICU = Intensive Care Unit*

***Notes****: We give the bacteriological results for the 59 episodes of VAP during the patient’s stay in intensive care.*

**Supplemental Table 3: Risk factors of non-ventilator-associated ICU-acquired pneumonia.**

| **Variable** | **Univariate *** | | **Multivariate **** | |
| --- | --- | --- | --- | --- |
|  | **sdHR (95%CI)** | **P value** | **sdHR (95%CI)** | **P value** |
| **Baseline characteristics** | | | | |
| Age | 1.01 [0.99; 1.03] | 0.51 |  |  |
| Male sex | 1.63 [0.82; 3.23] | 0.16 | 1.77 [0.87; 3.62] | 0.12 |
| BMI | 0.99 [0.95; 1.03] | 0.66 |  |  |
| Maximum SOFA score D1-D2 | 1.06 [0.96; 1.18] | 0.26 |  |  |
| Immunodeficiency | 2.16 [0.90; 5.21] | 0.09 | 2.01 [0.81; 4.96] | 0.13 |
| No decrease in consciousness D1-D2  (Glasgow Coma Scale = 15) | 0.33 [0.17; 0.63] | <.01 | 0.35 [0.16; 0.76] | <0.01 |
| MDR bacterial colonisation | 0.99 [0.25; 3.87] | 0.99 |  |  |
|  | | | | |
| **COPD Severity** | | | | |
| Very severe COPD | 0.13 [0.02; 1.05] | 0.06 | 0.14 [0.02; 1.1] | 0.06 |
|  | | | | |
| **Trigger of acute exacerbation of COPD** | | | | |
| Respiratory infection, n (%) | 1.72 [0.23; 12.58] | 0.81 |  |  |
| Non-infectious respiratory causes, n (%) | 1.78 [0.23; 13.88] |  |  |  |
| Cardiac and thromboembolic events, n (%) | 2.12 [0.22; 20.15] |  |  |  |
| Others, n (%) | 0 [0; 0] |  |  |  |
|  | | | | |
| **Therapeutics** | | | | |
| Corticosteroid therapy at admission | 0.74 [0.37; 1.46] | 0.38 |  |  |
| Antibiotic therapy at admission | 0.50 [0.26; 0.94] | 0.03 | 0.45 [0.23; 0.86] | 0.02 |
| Gastroprotective agents at admission | 1.41 [0.68; 2.93] | 0.35 |  |  |
| Enteral nutrition at admission | 2.17 [1.04; 4.50] | 0.04 | 1.18 [0.48; 2.87] | 0.72 |
|  | | | | |
| NIV at admission | 0.79 [0.41; 1.50] | 0.46 |  |  |
|  | | | | |
| **Hospitalisation before admission to ICU** | 1.58 [0.83; 2.99] | 0.16 | 1.67 [0.85; 3.27] | 0.13 |
| Days Hospitalisation before admission to ICU: 1-2 days | 1.86 [0.90; 3.83] | 0.09 |  |  |
| Days Hospitalisation before admission to ICU: 3-7 days | 1.24 [0.34; 4.45] | 0.74 |  |  |
| Days Hospitalisation before admission to ICU >7 days | 1.29 [0.43; 3.83] | 0.65 |  |  |

***Notes:*** ** = Univariate Fine and Gray competing risk model with stratification by centre. ** = Multivariate Fine and Gray competing risk model with stratification by centre. The prescription of corticosteroids therapy at admission was defined as a daily dose ≥ 0.5 mg/kg of prednisone or equivalent during the first 24 hours after admission to ICU.*

***Abbreviations:*** *sdHR = Sub-Distribution Hazard Ratio; BMI = Body Mass Index; SOFA Score = Sequential Organ Failure Assessment Score; COPD = Chronic Obstructive Pulmonary Disease, ICU = Intensive Care Unit.*

**Supplemental Table 4. Baseline characteristics and outcomes for patients with and without non-ventilator-associated ICU-acquired pneumonia according to the level of consciousness admitted in ICU for severe acute exacerbation of chronic obstructive pulmonary disease.**

|  | No decrease in consciousness Day 1- Day 2 (Glasgow Coma Scale = 15) (n=504) | | | Decrease in consciousness Day 1- Day 2  (Glasgow Coma Scale < 15) (n=340) | | |
| --- | --- | --- | --- | --- | --- | --- |
|  | No NV-ICU-AP (n=490) | NV-ICU-AP (n=14) | p Value | No NV-ICU-AP (n=312) | NV-ICU-AP (n=28) | p Value |
|  | Median [Q1; Q3] or n (percentage) | Median [Q1; Q3] or n (percentage) |  | Median [Q1; Q3] or n (percentage) | Median [Q1; Q3] or n (percentage) |  |
| **Baseline characteristics** | | | | | | |
| Age (years) | 69.7 [61.7; 77.2] | 76.4 [70.0; 78.3] | 0.04 | 72.2 [62.5; 79.4] | 71.0 [66.6; 75.2] | 0.49 |
| Male sex, n (%) | 311 (63.5) | 12 (85.7) | 0.09 | 188 (60.3) | 19 (67.9) | 0.43 |
| BMI (kg/m^2^) | 24.2 [20.6; 29.8] | 23.0 [21.0; 27.1] | 0.84 | 25.8 [21.1; 31.2] | 24.4 [21.2; 30.7] | 0.57 |
| SAPS II score | 31.0 [24.0; 38.0] | 37.5 [31.0; 40.0] | 0.05 | 38.0 [31.0; 48.0] | 39.0 [29.5; 50.5] | 0.90 |
| Maximum SOFA Day 1- Day 2 | 3.0 [2.0; 5.0] | 3.5 [3.0; 6.0] | 0.27 | 5.0 [4.0; 7.0] | 5.0 [4.0; 6.0] | 0.64 |
| Hospitalisation before ICU admission (yes), n (%) | 150 (30.6) | 7 (50.0) | 0.12 | 114 (36.5) | 15 (53.6) | 0.30 |
| Immunodeficiency (yes), n (%) | 41 (8.4) | 4 (28.6) | <0.01 | 33 (10.6) | 2 (7.1) | 0.57 |
| **COPD Severity** | | | | | | |
| Very Severe COPD, n (%) | 108 (22.0) | 0 | 0.05 | 65 (20.8) | 1 (3.6) | 0.03 |
| **Trigger of the acute exacerbation of COPD** | | | | | | |
| Respiratory infection, n (%) | 336 (68.6) | 10 (71.4) | 0.88 | 188 (60.3) | 19 (67.9) | 0.65 |
| Non-infectious respiratory causes, n (%) | 94 (19.2) | 3 (21.4) |  | 71 (22.8) | 5 (17.9) |  |
| Cardiac and thromboembolic events, n (%) | 38 (7.8) | 1 (7.1) |  | 25 (8.0) | 3 (10.7) |  |
| Others, n (%) | 22 (4.5) | 0 |  | 28 (9.0) | 1 (3.6) |  |
| **Therapeutic limitation** | | | | | | |
| Limitation of therapeutic effort at admission to ICU, n (%) | 28 (5.7) | 1 (7.1) | 0.82 | 35 (11.2) | 2 (7.1) | 0.51 |
| **Corticosteroid Therapy** | | | | | | |
| Use of corticosteroids therapy at admission, n (%) | 187 (38.2) | 4 (28.6) | 0.47 | 115 (36.9) | 8 (28.6) | 0.38 |
| **Antibiotic Therapy** | | | | | | |
| Use of antibiotic therapy at admission, n (%) | 339 (69.2) | 8 (57.1) | 0.34 | 222 (71.1) | 17 (60.7) | 0.25 |
| **Gastroprotective agents** | | | | | | |
| Use of gastroprotective agents at admission, n (%) | 226 (46.1) | 12 (85.7) | <0.01 | 185 (59.3) | 10 (35.7) | 0.02 |
| **Enteral Nutrition** | | | | | | |
| Use of enteral nutrition at admission, n (%) | 15 (3.1) | 1 (7.1) | 0.39 | 84 (26.9) | 9 (32.1) | 0.55 |
| **Lengths of stay** | | | | | | |
| ICU Length of stay (days) | 6.0 [5.0; 8.0] | 19.5 [10.0; 36.0] | <0.01 | 8.0 [5.0; 14.0] | 25.0 [15.0; 37.5] | <0.01 |
| Hospital Length of stay (days) | 17.0 [11.0; 27.0] | 37.0 [15.0; 54.0] | 0.02 | 21.0 [14.0; 37.0] | 37.0 [26.0; 61.0] | <0.01 |
| **Mortality** | | | | | | |
| ICU Mortality rate, n (%) | 25 (5.1) | 5 (35.7) | <0.01 | 48 (15.4) | 11 (39.3) | <0.01 |
| Hospital Mortality rate, n (%) | 50 (10.2) | 6 (42.9) | <0.01 | 73 (23.4) | 12 (42.8) | 0.02 |
| Mortality at Day 28, n (%) | 40 (8.2) | 3 (21.4) | 0.08 | 56 (17.9) | 7 (25.0) | 0.36 |
| **Non-ventilator-associated ICU-acquired pneumonia** | | | | | | |
| Day of first diagnosis of NV-ICU-AP (days in ICU) |  | 6.0 [4.0; 7.0] |  |  | 6.0 [4.0; 15.0] |  |
| NV-ICU-AP requiring intubation, n (%) |  | 10 (71.4) |  |  | 22 (78.6) |  |

***Abbreviations:*** *ICU = Intensive Care Unit; BMI = Body Mass Index, SAPS II = Simplified Acute Physiology Score II, SOFA Score = Sequential Organ Failure Assessment Score; COPD = Chronic Obstructive Pulmonary Disease, NV-ICU-AP = Non-ventilator-associated Intensive Care Unit Acquired Pneumonia*

**Supplemental Table 5: Risk factors for mortality up to day 28.**

| **Variable** | **Univariate *** | | **Multivariate **** | | **Multivariate ***** | |
| --- | --- | --- | --- | --- | --- | --- |
|  | **HR (95%CI)** | **P value** | **HR (95%CI)** | **P value** | **HR (95%CI)** | **P value** |
| **Baseline characteristics** | | | | | | |
| Age | 1.06 [1.03; 1.08] | <.01 | 1.06 [1.03; 1.09] | <.01 | 1.06 [1.03; 1.09] | <.01 |
| Male sex | 1.01 [0.64; 1.60] | 0.96 |  |  |  |  |
| BMI | 1.02 [0.99; 1.05] | 0.25 |  |  |  |  |
| Maximum SOFA score D1-D2 | 1.29 [1.19; 1.39] | <.01 | 1.25 [1.12; 1.38] | <.01 | 1.25 [1.13; 1.34] | <.01 |
| Immunodeficiency | 2.61 [1.53; 4.45] | <.01 | 2.31 [1.27; 4.21] | <.01 | 2.27 [1.25; 4.13] | <.01 |
| Minimum Glasgow Coma Scale Day-1- Day-2 | 0.90 [0.86; 0.94] | <.01 | 0.95 [0.90; 1.00] | 0.06 | 0.95 [0.90; 1.00] | 0.08 |
|  | | | | | | |
| **COPD Severity** | | | | | | |
| Very severe COPD | 0.67 [0.34; 1.32] | 0.25 |  |  |  |  |
|  | | | | | | |
| **Trigger of acute exacerbation of COPD** | | | | | | |
| Respiratory infection, n (%) | 0.40 [0.20; 0.80] | <.01 | 0.62 [0.30; 1.26] | 0.16 | 0.61 [0.29; 1.23] | 0.14 |
| Non-infectious respiratory causes, n (%) | 0.21 [0.08; 0.52] |  | 0.34 [0.13; 0.87] |  | 0.32 [0.13; 0.89] |  |
| Cardiac and thromboembolic events, n (%) | 0.58 [0.23; 1.45] |  | 0.60 [0.24; 1.54] |  | 0.61 [0.23; 1.50] |  |
| Others, n (%) (reference) | 1 [.; .] |  | 1 [.; .] |  | 1 [.; .] |  |
|  | | | | | | |
| **Therapeutics** | | | | | | |
| corticosteroid therapy at admission | 1.16 [0.75; 1.82] | 0.50 |  |  |  |  |
| antibiotic therapy at admission | 1.43 [0.86; 2.39] | 0.17 |  |  |  |  |
|  | | | | | | |
| NIV at admission | 1.05 [0.65; 1.68] | 0.85 |  |  |  |  |
|  | | | | | | |
| **Hospitalisation before admission to ICU** | 2.38 [1.53; 3.68] | <.01 | 1.92 [1.23; 3.00] | <.01 | 1.90 [1.21; 2.96] | <.01 |
|  | | | | | | |
| **NV-ICU-AP** | 2.90 [1.35; 6.23] | <.01 | 3.03 [1.36; 6.73] | <.01 |  |  |
| **NV-ICU-AP requiring intubation** | 3.39 [1.51; 7.63] | <.01 |  |  | 4.23 [1.88; 9.55] | <.01 |

***Notes:*** ** = Univariate Cox model with stratification by centre, NV-ICU-AP treated as time-dependent variable. NV-ICU-AP requiring intubation also treated as time-dependent variable. ** = Multivariate Cox model with stratification by centre, using a stepwise selection and NV-ICU-AP treated as time-dependent variable. *** = Multivariate Cox model with stratification by centre, using a stepwise selection and NV-ICU-AP requiring intubation treated as time-dependent variable. The prescription of corticosteroids therapy at admission was defined as a daily dose ≥ 0.5 mg/kg of prednisone or equivalent during the first 24 hours after admission to ICU.*

***Abbreviations:*** *HR = Hazard Ratio; BMI = Body Mass Index; SOFA Score = Sequential Organ Failure Assessment Score; COPD = Chronic Obstructive Pulmonary Disease, ICU = Intensive Care Unit. NV-ICU-AP= Non-ventilator-associated ICU-acquired pneumonia.*

**Supplemental Table 6: Risk factors for intubation and invasive mechanical ventilation for patients at risk of non-ventilator-associated ICU-acquired pneumonia. Univariate Analysis.**

| **Variable** | Intubation (n=187) | | Died in ICU (no intubation) (n= 32) | | Discharged from ICU (no intubation) (n=619) | |
| --- | --- | --- | --- | --- | --- | --- |
|  | **csHR (95%CI)** | **P value** | **csHR (95%CI)** | **P value** | **csHR (95%CI)** | **P value** |
| **Baseline characteristics** | | | | | | |
| Age | 0.99 [0.98; 1.00] | 0.15 | 1.11 [1.06; 1.16] | <.01 | 0.99 [0.98; 1.0] | 0.01 |
| Male sex | 1.13 [0.83; 1.53] | 0.45 | 1.27 [0.57; 2.85] | 0.55 | 0.85 [0.72; 1.01] | 0.07 |
| BMI | 0.99 [0.98; 1.01] | 0.65 | 0.96 [0.91; 1.02] | 0.20 | 1.00 [0.99; 1.01] | 0.85 |
| Maximum SOFA score D1-D2 | 1.25 [1.19; 1.31] | <.01 | 1.2 [1.05; 1.36] | <.01 | 0.95 [0.92; 0.98] | <.01 |
| Immunodeficiency | 1.83 [1.20; 2.77] | <.01 | 1.80 [0.65; 4.96] | 0.25 | 0.58 [0.42; 0.80] | <.01 |
| Minimum Glasgow Coma Scale Day-1- Day-2 | 0.77 [0.75; 0.80] | <.01 | 0.97 [0.88; 1.08] | 0.58 | 1.07 [1.04; 1.11] | <.01 |
|  | | | | | | |
| **COPD Severity** | | | | | | |
| Very severe COPD | 0.58 [0.37; 0.92] | 0.02 | 2.58 [1.06; 6.32] | 0.04 | 0.99 [0.80; 1.22] | 0.89 |
|  | | | | | | |
| **Trigger of acute exacerbation of COPD** | | | | | | |
| Respiratory infection | 0.73 [0.42; 1.29] | 0.70 | 0.23 [0.09; 0.58] | <.01 | 1.41 [0.97; 2.05] | 0.25 |
| Non-infectious respiratory causes | 0.84 [0.45; 1.57] |  | 0.04 [0.01; 0.33] |  | 1.49 [0.99; 2.24] |  |
| Cardiac and thromboembolic events | 0.79 [0.37; 1.66] |  | 0.14 [0.02; 1.14] |  | 1.53 [0.97; 2.43] |  |
| Others (reference) | - |  | - |  | - |  |
|  | | | | | | |
| **Therapeutics** | | | | | | |
| corticosteroids therapy at admission | 1.51 [1.13; 2.03] | <.01 | 0.91 [0.43; 1.90] | 0.80 | 1.11 [0.94; 1.32] | 0.21 |
| antibiotic therapy at admission | 1.39 [0.99; 1.95] | 0.06 | 2.12 [0.85; 5.31] | 0.11 | 1.01 [0.85; 1.20] | 0.91 |
|  | | | | | | |
| NIV at admission | 0.64 [0.47; 0.87] | <.01 | 2.79 [1.00; 7.74] | 0.05 | 0.89 [0.74; 1.06] | 0.20 |
|  | | | | | | |
| **Therapeutic limitation** | | | | | | |
| Limitation of therapeutic effort at admission to ICU | 0.97 [0.55; 1.73] | 0.93 | 6.51 [2.93; 14.44] | <.01 | 1.00 [0.73; 1.38] | 0.99 |
|  | | | | | | |
| **Hospitalisation before admission ICU** | 1.11 [0.82; 1.50] | 0.51 | 1.71 [0.84; 3.48] | 0.14 | 0.84 [0.70; 0.99] | 0.04 |
| 1-2 days Hospitalisation before admission to ICU | 1.24 [0.85; 1.79] | 0.26 | 2.08 [0.88; 4.92] | 0.09 | 0.90 [0.73; 1.12] | 0.36 |
| 3-7 days Hospitalisation before admission to ICU | 0.91 [0.5; 1.67] | 0.77 | 1.54 [0.43; 5.52] | 0.51 | 0.93 [0.67; 1.29] | 0.66 |
| Days Hospitalisation before admission to ICU >7 days | 1.02 [0.62; 1.67] | 0.95 | 1.30 [0.42; 4.01] | 0.65 | 0.66 [0.48; 0.89] | <.01 |
|  | | | | | | |
| **NV-ICU-AP** | 5.10 [2.72; 9.55] | <.01 | . | . | 0.35 [0.15; 0.81] | 0.01 |

***Notes:*** *Univariate cause-specific Cox proportional hazards model with competing risks extension with stratification by centre and ICU-AP treated as time-dependent variable. 6 still alive in ICU at D28 without Intubation. There was not NV-ICU-AP event for patients who died in ICU without intubation. The prescription of corticosteroids therapy at admission was defined as a daily dose ≥ 0.5 mg/kg of prednisone or equivalent during the first 24 hours after admission in ICU.*

***Abbreviations:*** *csHR = cause-specific Hazard Ratio; BMI = Body Mass Index; SOFA Score = Sequential Organ Failure Assessment Score; COPD = Chronic Obstructive Pulmonary Disease, ICU = Intensive Care Unit. NV-ICU-AP= Non-ventilator-associated ICU-acquired pneumonia.*

**Supplemental Table 7: Risk factors of intubation and invasive mechanical ventilation for patients at risk of non-ventilator-associated ICU-acquired pneumonia. Multivariate Analysis.**

| **Variable** | Intubation (n=187) | | Died in ICU (no intubation) (n= 32) | | Discharge of ICU (no intubation) (n=619) | |
| --- | --- | --- | --- | --- | --- | --- |
|  | **csHR (95%CI)** | **P value** | **csHR (95%CI)** | **P value** | **csHR (95%CI)** | **P value** |
| **Baseline characteristics** | | | | | | |
| Age |  |  | 1.09 [1.04; 1.15] | <.01 | 0.99 [0.98; 1.00] | <.01 |
| Male sex |  |  | 2.10 [0.84; 5.26] | 0.11 | 0.83 [0.70; 0.99] | 0.04 |
| BMI |  |  | 0.95 [0.90; 1.01] | 0.07 |  |  |
| Maximum SOFA score D1-D2 | 1.07 [1.01; 1.14] | 0.02 | 1.13 [0.98; 1.30] | 0.09 |  |  |
| Immunodeficiency |  |  |  |  | 0.57 [0.41; 0.79] | <.01 |
| Minimum Glasgow Coma Scale Day-1- Day-2 | 0.78 [0.75; 0.81] | <.01 |  |  | 1.07 [1.03; 1.10] | <.01 |
|  | | | | | | |
| **COPD Severity** | | | | | | |
| Very severe COPD | 0.61 [0.38; 0.99] | 0.12 | 2.13 [0.85; 5.35] | 0.11 |  | - |
|  | | | | | | |
| **Trigger of acute exacerbation of COPD** | | | | | | |
| Respiratory infection |  | - | 0.28 [0.11; 0.72] | 0.02 |  | - |
| Non-infectious respiratory causes |  |  | 0.08 [0.01; 0.68] |  |  |  |
| Cardiac and thromboembolic events |  |  | 0.14 [0.02; 1.26] |  |  |  |
| Others (reference) | - |  | - |  | - |  |
|  | | | | | | |
| **Therapeutics** | | | | | | |
| corticosteroids therapy at admission | 1.55 [1.14; 2.11] | <.01 |  |  |  |  |
| antibiotic therapy at admission | 1.43 [0.99; 2.06] | 0.06 |  |  |  |  |
|  | | | | | | |
| NIV at admission | 0.80 [0.58; 1.12] | 0.19 | 2.28 [0.81; 6.39] | 0.12 | 0.88 [0.73; 1.05] | 0.15 |
|  | | | | | | |
| **Therapeutic limitation** | | | | | | |
| Limitation of therapeutic effort at admission to ICU |  |  | 3.52 [1.29; 9.61] | 0.01 |  |  |
|  | | | | | | |
| **Hospitalisation before admission to ICU** |  |  | 1.86 [0.86; 4.06] | 0.17 | 0.88 [0.74; 1.05] | 0.16 |
|  | | | | | | |
| **NV-ICU-AP** | 5.00 [2.54; 9.85] | <.01 | . | . | 0.40 [0.17; 0.91] | 0.03 |

***Notes:***  *Multivariate cause-specific Cox proportional hazards model with competing risks extension with stratification by centre and ICU-AP treated as time-dependent variable. 6 still alive in IUC at D-28 without Intubation. There was not NV-ICU-AP event for patients Died in ICU without intubation. The prescription of corticosteroids therapy at admission was defined as a daily dose ≥ 0.5 mg/kg of prednisone or equivalent during the first 24 hours after admission in ICU.*

***Abbreviations:*** *csHR = cause-specific Hazard Ratio; BMI = Body Mass Index; SOFA Score = Sequential Organ Failure Assessment Score; COPD = Chronic Obstructive Pulmonary Disease, ICU = Intensive Care Unit. NV-ICU-AP= Non-ventilator-associated ICU-acquired pneumonia.*

**Supplemental Table 8: Risk factors associated at length of stay in ICU for patients at risk of non-ventilator-associated ICU-acquired pneumonia. Univariate Analysis.**

| **Variable** | Discharged from ICU (n=715) | | | Died in ICU (n=75) | | |
| --- | --- | --- | --- | --- | --- | --- |
|  | **Increase LOS** | **Decrease LOS** |  | **Increase LOS** | **Decrease LOS** |  |
|  | **csHR (95%CI)** | **csHR (95%CI)** | **P value** | **csHR (95%CI)** | **csHR (95%CI)** | **P value** |
| **Baseline characteristics** | | | | | | |
| Age | 0.99 [0.98; 1.00] | - | 0.03 | - | 1.07 [1.04; 1.10] | <.01 |
| Male sex | 0.86 [0.73; 1.01] | - | 0.06 | - | 1.43 [0.84; 2.44] | 0.19 |
| BMI | 1 [0.99; 1.01] | - | 0.98 | 0.99 [0.96; 1.02] | - | 0.57 |
| Maximum SOFA score D1-D2 | 0.91 [0.88; 0.93] | - | <.01 | - | 1.20 [1.11; 1.30] | <.01 |
| Immunodeficiency | 0.54 [0.41; 0.72] | - | <.01 | - | 1.13 [0.58; 2.18] | 0.72 |
| Minimum Glasgow Coma Scale Day-1- Day-2 | - | 1.11 [1.09; 1.14] | <.01 | 0.97 [0.92; 1.01] |  | 0.17 |
|  | | | | | | |
| **COPD Severity** | | | | | | |
| Very severe COPD | - | 1.13 [0.93; 1.37] | 0.21 | - | 1.24 [0.67; 2.30] | 0.50 |
|  | | | | | | |
| **Trigger of acute exacerbation of COPD** | | | | | | |
| Respiratory infection | - | 1.34 [0.95; 1.88] | 0.32 | 0.45 [0.22; 0.90] | - | 0.02 |
| Non-infectious respiratory causes | - | 1.39 [0.97; 2.00] |  | 0.20 [0.08; 0.55] | - |  |
| Cardiac and thromboembolic events | - | 1.23 [0.80; 1.88] |  | 0.47 [0.17; 1.29] | - |  |
| Others (reference) | - | - |  | - | - |  |
|  | | | | | | |
| **Therapeutics** | | | | | | |
| corticosteroids therapy at admission | 1.00 [0.85; 1.16] | - | 0.97 | 0.94 [0.58; 1.52] | - | 0.81 |
| antibiotic therapy at admission | 0.93 [0.79; 1.09] | - | 0.38 | - | 1.66 [0.94; 2.92] | 0.08 |
|  | | | | | | |
| NIV at admission | - | 1.1 [0.93; 1.29] | 0.25 | - | 1.00 [0.61; 1.64] | 0.99 |
|  | | | | | | |
| **Therapeutic limitation** | | | | | | |
| Limitation of therapeutic effort at admission to ICU | 0.90 [0.66; 1.21] | - | 0.48 | - | 3.09 [1.70; 5.61] | <.01 |
|  | | | | | | |
| **Hospitalisation before admission to ICU** | 0.83 [0.71; 0.97] | - | 0.02 | - | 1.52 [0.95; 2.43] | 0.08 |
|  | | | | | | |
| **NV-ICU-AP** | 0.37 [0.22; 0.64] | - | <.01 | - | 1.44 [0.67; 3.12] | 0.35 |

***Notes:*** *Assessment of the impact of ICU-AP on length of stay (by the measure of the instantaneous risk of alive discharge of ICU) using cause-specific Cox proportional hazards model with competing risks with stratification by center and ICU-AP treated as time-dependent variable. 54 still alive in ICU at D-28. The prescription of corticosteroids therapy at admission was defined as a daily dose ≥ 0.5 mg/kg of prednisone or equivalent during the first 24 hours after admission in ICU.*

***Abbreviations:*** *csHR = cause-specific Hazard Ratio; LOS= Length of stay; BMI = Body Mass Index; SOFA Score = Sequential Organ Failure Assessment Score; COPD = Chronic Obstructive Pulmonary Disease, ICU = Intensive Care Unit. NV-ICU-AP= Non-ventilator-associated ICU-acquired pneumonia.*

**Supplemental Table 9: Risk factors associated with length of stay in ICU for patients at risk of non-ventilator-associated ICU-acquired pneumonia. Multivariate Analysis.**

| **Variable** | Discharged from ICU (n=715) | | | Died in ICU (n=75) | | |
| --- | --- | --- | --- | --- | --- | --- |
|  | **Increase LOS** | **Decrease LOS** |  | **Increase LOS** | **Decrease LOS** |  |
|  | **csHR (95%CI)** | **csHR (95%CI)** | **P value** | **csHR (95%CI)** | **csHR (95%CI)** | **P value** |
| **Baseline characteristics** | | | | | | |
| Age | 0.99 [0.98; 1.00] | - | <.01 | - | 1.07 [1.04; 1.10] | <.01 |
| Male sex | 0.87 [0.74; 1.02] | - | 0.09 | - | 1.54 [0.87; 2.72] | 0.14 |
| BMI | - | - | - | 0.97 [0.94; 1.01] | - | 0.15 |
| Maximum SOFA score D1-D2 | 0.97 [0.94; 1.0] | - | 0.08 | - | 1.20 [1.10; 1.31] | <.01 |
| Immunodeficiency | 0.59 [0.44; 0.79] | - | <.01 | - | - | - |
| Minimum Glasgow Coma Scale Day-1- Day-2 |  | 1.11 [1.08; 1.14] | <.01 |  |  |  |
|  | | | | | | |
| **COPD Severity** | | | | | | |
| Very severe COPD | - | - | - | - | 1.70 [0.90; 3.23] | 0.10 |
|  | | | | | | |
| **Trigger of acute exacerbation of COPD** | | | | | | |
| Respiratory infection | - | - | - | - | - | - |
| Non-infectious respiratory causes | - | - |  | - | - |  |
| Cardiac and thromboembolic events | - | - |  | - | - |  |
| Others (reference) | - | - |  | - | - |  |
|  | | | | | | |
| **Therapeutics** | | | | | | |
| corticosteroids therapy at admission | - | - | - | - | - | - |
| antibiotic therapy at admission | - | - | - | - | - | - |
|  | | | | | | |
| NIV at admission | - | - | - | - | - | - |
|  | | | | | | |
| **Therapeutic limitation** | | | | | | |
| Limitation of therapeutic effort at admission to ICU | - | - | - | - | 2.15 [1.13; 4.08] | 0.02 |
|  | | | | | | |
| **Hospitalisation before admission to ICU** | 0.90 [0.76; 1.06] | - | 0.20 | - | 1.44 [0.88; 2.37] | 0.15 |
|  | | | | | | |
| **NV-ICU-AP** | 0.38 [0.22; 0.66] | - | <.01 | - | 2.35 [1.03; 5.38] | 0.04 |

***Notes:***  *Assessment of the impact of ICU-AP on length of stay (by the measure of the instantaneous risk of alive discharge of ICU) using cause-specific Cox proportional hazards model with competing risks with stratification by centre and ICU-AP treated as time-dependent variable, using a stepwise selection method from the univariate model. 54 still alive in IUC at D-28. The prescription of corticosteroids therapy at admission was defined as a daily dose ≥ 0.5 mg/kg of prednisone or equivalent during the first 24 hours after admission in ICU.*

***Abbreviations:*** *csHR = cause-specific Hazard Ratio; LOS= Length of stay; BMI = Body Mass Index; SOFA Score = Sequential Organ Failure Assessment Score; COPD = Chronic Obstructive Pulmonary Disease, ICU = Intensive Care Unit. NV-ICU-AP= Non-ventilator-associated ICU-acquired pneumonia.*

**Supplemental Table 10: Impact of antibiotic treatment on day 28 mortality.**

| **Variable** | **Univariate *** | | **Multivariate **** | | **Multivariate ***** | | **Multivariate ****** | |
| --- | --- | --- | --- | --- | --- | --- | --- | --- |
|  | **HR (95%CI)** | **P value** | **HR (95%CI)** | **P value** | **HR (95%CI)** | **P value** | **HR (95%CI)** | **P value** |
| **Baseline characteristics** | | | | | | | | |
| Age | 1.14 [0.95; 1.37] | 0.16 | 1.17 [0.94; 1.46] | 0.15 | 1.57 [0.72; 3.42] | 0.26 | 1.13 [0.94; 1.36] | 0.16 |
| Male sex | 3.13 [0.37; 26.20] | 0.29 |  |  |  |  |  |  |
| BMI | 1.04 [0.95; 1.14] | 0.40 |  |  |  |  |  |  |
| Maximum SOFA score D1-D2 | 0.88 [0.58; 1.34] | 0.54 |  |  | 0.52 [0.24; 1.11] | 0.09 |  |  |
| Immunodeficiency | 1.34 [0.17; 10.62] | 0.78 |  |  |  |  |  |  |
| Minimum Glasgow Coma Scale Day-1- Day-2 | 0.99 [0.82; 1.20] | 0.92 |  |  | 0.63 [0.31; 1.28] | 0.20 |  |  |
|  | | | | | | | | |
| **COPD Severity** | | | | | | | | |
| Very severe COPD | - | 0.99 |  |  |  |  |  |  |
|  | | | | | | | | |
| **Trigger of acute exacerbation of COPD** | | | | | | | | |
| Respiratory infection, n (%) | 0.41 [0.02; 6.62] | 0.93 |  |  |  |  |  |  |
| Non-infectious respiratory causes, n (%) | - |  |  |  |  |  |  |  |
| Cardiac and thromboembolic events, n (%) | 0.50 [0.23; 1.45] |  |  |  |  |  |  |  |
| Others, n (%) (reference) | 1 [.; .] |  |  |  |  |  |  |  |
|  | | | | | | | | |
| **Therapeutics** | | | | | | | | |
| corticosteroids therapy at admission | 1.08 [0.18; 6.44] | 0.93 |  |  |  |  |  |  |
| antibiotic therapy at admission | 0.55 [0.11; 2.64] | 0.45 |  |  |  |  |  |  |
|  | | | | | | | | |
| NIV at admission | 0.41 [0.07; 2.53] | 0.34 |  |  |  |  |  |  |
|  | | | | | | | | |
| **Hospitalisation before admission ICU** | 1.11 [0.23; 5.37] | 0.89 |  |  |  |  |  |  |
|  | | | | | | | | |
| **Inappropriate antibiotic treatment** | 0.40 [0.07; 2.16] | 0.29 | 0.36 [0.06; 2.20] | 0.27 |  |  |  |  |
| **Time to appropriate antibiotic treatment (days)** | 0.56 [0.21; 1.49] | 0.25 |  |  | 0.25 [0.02; 2.86] | 0.26 |  |  |
| **MDR bacteria detected** | 0.35 [0.04; 3.18] | 0.35 |  |  |  |  | 0.36 [0.04; 3.53] | 0.38 |

***Notes:*** ** = Univariate Cox model with stratification by centre. With the 33 NV-ICU-APs with microbiologically documentation and no therapeutic limitation at admission in ICU. ** = Multivariate Cox model with stratification by centre, using a stepwise selection. *** = Multivariate Cox model with stratification by centre, using a stepwise selection. **** = Multivariate Cox model with stratification by centre, using a stepwise selection. The prescription of corticosteroids therapy at admission was defined as a daily dose ≥ 0.5 mg/kg of prednisone or equivalent during the first 24 hours after admission in ICU.*

***Abbreviations:*** *HR = Hazard Ratio; BMI = Body Mass Index; SOFA Score = Sequential Organ Failure Assessment Score; COPD = Chronic Obstructive Pulmonary Disease, ICU = Intensive Care Unit. NV-ICU-AP= Non-ventilator-associated ICU-acquired pneumonia. MDR= Multi Drug Resistance.*

**Supplemental Table 11: Impact of antibiotic treatment on intubation. Univariate Analysis.**

| **Variable** | Intubation (n=28) | | Died in ICU (no intubation) (n= 1) | | Discharged from ICU (no intubation) (n=5) | |
| --- | --- | --- | --- | --- | --- | --- |
|  | **csHR (95%CI)** | **P value** | **csHR (95%CI)** | **P value** | **csHR (95%CI)** | **P value** |
| **Baseline characteristics** | | | | | | |
| Age | 0.98 [0.92; 1.04] | 0.56 | 0.82 [0.57; 1.19] | 0.82 | 1.02 [0.88; 1.19] | 0.76 |
| Male sex | 0.75 [0.32; 1.73] | 0.73 | . | . | 0.38 [0.06; 2.39] | 0.30 |
| BMI | 1.01 [0.95; 1.07] | 0.76 | 0.78 [0.42; 1.44] | 0.43 | 1.06 [0.94; 1.20] | 0.33 |
| Maximum SOFA score D1-D2 | 1.19 [1.00; 1.42] | 0.04 | 1.05 [0.47; 2.32] | 0.91 | 0.83 [0.54; 1.27] | 0.39 |
| Immunodeficiency | 2.04 [0.75; 5.53] | 0.16 | . | . | . | . |
| Minimum Glasgow Coma Scale Day-1- Day-2 | 0.83 [0.75; 0.92] | <.01 | . | . | 1.21 [0.77; 1.90] | 0.41 |
|  | | | | | | |
| **COPD Severity** | | | | | | |
| Very severe COPD | 1.78 [0.24; 13.47] | 0.58 | . | . | . | . |
|  | | | | | | |
| **Trigger of acute exacerbation of COPD** | | | | | | |
| Respiratory infection | 1.70 [0.22; 12.95] | 0.49 | . | . | 0.68 [0.01; 5.55] | 0.72 |
| Non-infectious respiratory causes | 1.17 [0.13; 10.22] |  | . |  | . |  |
| Cardiac and thromboembolic events | 4.24 [0.36; 50.88] |  | . |  | . |  |
| Others (reference) | - |  | - |  | - |  |
|  | | | | | | |
| **Therapeutics** | | | | | | |
| corticosteroids therapy at admission | 2.15 [0.94; 4.90] | 0.07 | . | . | 1.87 [0.15; 22.53] | 0.62 |
| antibiotic therapy at admission | 1.31 [0.60; 2.86] | 0.50 | . | . | 3.31 [0.37; 29.81] | 0.29 |
|  | | | | | | |
| NIV at admission | 1.15 [0.51; 2.57] | 0.74 | . | . | . | . |
|  | | | | | | |
| **Therapeutic limitation** | | | | | | |
| Limitation of therapeutic effort at admission to ICU | 3.16 [0.66; 15.01] | 0.15 | . | . | . | . |
|  | | | | | | |
| **Hospitalisation before admission to ICU** | 1.83 [0.84; 3.95] | 0.13 | . | . | 0.48 [0.05; 4.41] | 0.13 |
|  | | | | | | |
| **Inappropriate antibiotic treatment** | 1.06 [0.50; 2.24] | 0.88 | . | . | 0.72 [0.12; 4.32] | 0.72 |
| **Time to appropriate antibiotic treatment** | 0.91 [0.68; 1.22] | 0.52 | . | . | 1.00 [0.54; 1.85] | 0.99 |
| **MDR bacteria detected** | 0.73 [0.33; 1.63] | 0.45 |  |  | 0.88 [0.14; 5.40] | 0.88 |

***Notes:*** *Univariate cause-specific Cox proportional hazards model with competing risks extension. With the 36 NV-ICU-APs with microbiologically documentation. 2 still alive in ICU at D-28 without Intubation. The prescription of corticosteroids therapy at admission was defined as a daily dose ≥ 0.5 mg/kg of prednisone or equivalent during the first 24 hours after admission in ICU.*

***Abbreviations:*** *csHR = cause-specific Hazard Ratio; BMI = Body Mass Index; SOFA Score = Sequential Organ Failure Assessment Score; COPD = Chronic Obstructive Pulmonary Disease, ICU = Intensive Care Unit. NV-ICU-AP= Non-ventilator-associated ICU-acquired pneumonia. MDR= Multi Drug Resistance.*

**Supplemental Table 12: Impact of inappropriate antibiotic treatment on intubation. Multivariate Analysis.**

| **Variable** | Intubation (n=28) | | Died in ICU (no intubation) (n= 1) | | Discharged from ICU (no intubation) (n=5) | |
| --- | --- | --- | --- | --- | --- | --- |
|  | **csHR (95%CI)** | **P value** | **csHR (95%CI)** | **P value** | **csHR (95%CI)** | **P value** |
| **Baseline characteristics** | | | | | | |
| Age | . | . | . | . | . | . |
| Male sex | . | . | . | . | . | . |
| BMI | . | . | . | . | . | . |
| Maximum SOFA score D1-D2 | 1.23 [1.00; 1.52] | 0.05 | . | . | . | . |
| Immunodeficiency | 3.76 [1.11; 12.75] | 0.03 | . | . | . | . |
| Minimum Glasgow Coma Scale Day-1- Day-2 | 0.80 [0.71; 0.91] | <.01 | . | . | . | . |
|  | | | | | | |
| **COPD Severity** | | | | | | |
| Very severe COPD | . | . | . | . | . | . |
|  | | | | | | |
| **Trigger of acute exacerbation of COPD** | | | | | | |
| Respiratory infection | . | . | . | . | . | . |
| Non-infectious respiratory causes | . |  | . |  | . |  |
| Cardiac and thromboembolic events | . |  | . |  | - |  |
| Others (reference) | - |  | - |  | - |  |
|  | | | | | | |
| **Therapeutics** | | | | | | |
| corticosteroids therapy at admission | 2.74 [0.96; 7.80] | 0.05 | . | . | . | . |
| antibiotic therapy at admission | . | . | . | . | . | . |
|  | | | | | | |
| NIV at admission | . | . | . | . | . | . |
|  | | | | | | |
| **Therapeutic limitation** | | | | | | |
| Limitation of therapeutic effort at admission to ICU | 4.14 [0.67; 25.71] | 0.12 | . | . | . | . |
|  | | | | | | |
| **Hospitalisation before admission to ICU** | . | . | . | . | . | . |
|  | | | | | | |
| **Inappropriate antibiotic treatment** | 0.76 [0.03; 1.87] | 0.55 | . | . | 0.68 [0.1; 4.13] | 0.68 |

***Notes:***  *Multivariate cause-specific Cox proportional hazards model with competing risks extension. With the 36 NV-ICU-APs with microbiologically documentation. 2 still alive in ICU at D-28 without Intubation. The prescription of corticosteroids therapy at admission was defined as a daily dose ≥ 0.5 mg/kg of prednisone or equivalent during the first 24 hours after admission in ICU.*

***Abbreviations:*** *csHR = cause-specific Hazard Ratio; BMI = Body Mass Index; SOFA Score = Sequential Organ Failure Assessment Score; COPD = Chronic Obstructive Pulmonary Disease, ICU = Intensive Care Unit. NV-ICU-AP= Non-ventilator-associated ICU-acquired pneumonia. MDR= Multi Drug Resistance.*

**Supplemental Table 13: Impact of time to adequate antibiotic treatment on intubation. Multivariate Analysis.**

| **Variable** | Intubation (n=28) | | Died in ICU (no intubation) (n= 1) | | Discharged from ICU (no intubation) (n=5) | |
| --- | --- | --- | --- | --- | --- | --- |
|  | **csHR (95%CI)** | **P value** | **csHR (95%CI)** | **P value** | **csHR (95%CI)** | **P value** |
| **Baseline characteristics** | | | | | | |
| Age | . | . | . | . | . | . |
| Male sex | . | . | . | . | . | . |
| BMI | . | . | . | . | . | . |
| Maximum SOFA score D1-D2 | 1.21 [0.97; 1.50] | 0.09 | . | . | . | . |
| Immunodeficiency | 3.58 [1.05; 12.14] | 0.04 | . | . | . | . |
| Minimum Glasgow Coma Scale Day-1- Day-2 | 0.80 [0.70; 0.90] | <.01 | . | . | . | . |
|  | | | | | | |
| **COPD Severity** | | | | | | |
| Very severe COPD | . | . | . | . | . | . |
|  | | | | | | |
| **Trigger of acute exacerbation of COPD** | | | | | | |
| Respiratory infection | . | . | . | . | . | . |
| Non-infectious respiratory causes | . |  | . |  | . |  |
| Cardiac and thromboembolic events | . |  | . |  | - |  |
| Others (reference) | - |  | - |  | - |  |
|  | | | | | | |
| **Therapeutics** | | | | | | |
| corticosteroids therapy at admission | 2.84 [0.98; 8.25] | 0.05 | . | . | . | . |
| antibiotic therapy at admission | . | . | . | . | . | . |
|  | | | | | | |
| NIV at admission | . | . | . | . | . | . |
|  | | | | | | |
| **Therapeutic limitation** | | | | | | |
| Limitation of therapeutic effort at admission to ICU | 4.42 [0.74; 26.57] | 0.10 | . | . | . | . |
|  | | | | | | |
| **Hospitalisation before admission ICU** | . | . | . | . | . | . |
|  | | | | | | |
| **Time to adequate** **antibiotic treatment** | 0.81 [0.57; 1.16] | 0.25 | . | . | 1.06 [0.53; 2.11] | 0.87 |

***Notes:***  *Multivariate cause-specific Cox proportional hazards model with competing risks extension. With the 36 NV-ICU-APs with microbiologically documentation. 2 still alive in ICU at D-28 without Intubation. The prescription of corticosteroids therapy at admission was defined as a daily dose ≥ 0.5 mg/kg of prednisone or equivalent during the first 24 hours after admission in ICU.*

***Abbreviations:*** *csHR = cause-specific Hazard Ratio; BMI = Body Mass Index; SOFA Score = Sequential Organ Failure Assessment Score; COPD = Chronic Obstructive Pulmonary Disease, ICU = Intensive Care Unit. NV-ICU-AP= Non-ventilator-associated ICU-acquired pneumonia.*

**Supplemental Table 14: Impact of multi drug resistance bacteria microbiological documentation on intubation. Multivariate Analysis.**

| **Variable** | Intubation (n=28) | | Died in ICU (no intubation) (n= 1) | | Discharged from ICU (no intubation) (n=5) | |
| --- | --- | --- | --- | --- | --- | --- |
|  | **csHR (95%CI)** | **P value** | **csHR (95%CI)** | **P value** | **csHR (95%CI)** | **P value** |
| **Baseline characteristics** | | | | | | |
| Age | . | . | . | . | . | . |
| Male sex | . | . | . | . | . | . |
| BMI | . | . | . | . | . | . |
| Maximum SOFA scoreD1-D2 | 1.22 [0.99; 1.50] | 0.06 | . | . | . | . |
| Immunodeficiency | 4.63 [1.35; 15.86] | 0.01 | . | . | . | . |
| Minimum Glasgow Coma Scale Day-1- Day-2 | 0.81 [0.72; 0.91] | <.01 | . | . | . | . |
|  | | | | | | |
| **COPD Severity** | | | | | | |
| Very severe COPD | . | . | . | . | . | . |
|  | | | | | | |
| **Trigger of acute exacerbation of COPD** | | | | | | |
| Respiratory infection | . | . | . | . | . | . |
| Non-infectious respiratory causes | . |  | . |  | . |  |
| Cardiac and thromboembolic events | . |  | . |  | - |  |
| Others (reference) | - |  | - |  | - |  |
|  | | | | | | |
| **Therapeutics** | | | | | | |
| corticosteroids therapy at admission | 2.61 [0.90; 7.57] | 0.08 | . | . | . | . |
| antibiotic therapy at admission | . | . | . | . | . | . |
|  | | | | | | |
| NIV at admission | . | . | . | . | . | . |
|  | | | | | | |
| **Therapeutic limitation** | | | | | | |
| Limitation of therapeutic effort at admission to ICU | 4.57 [0.75; 27.86] | 0.10 | . | . | . | . |
|  | | | | | | |
| **Hospitalisation before admission to ICU** | . | . | . | . | . | . |
|  | | | | | | |
| **MDR bacteria detected** | 0.61 [0.26; 1.44] | 0.26 | . | . | 1.08 [0.15; 7.68] | 0.79 |

***Notes:***  *Multivariate cause-specific Cox proportional hazards model with competing risks extension. With the 36 NV-ICU-APs with microbiologically documentation. 2 still alive in IUC at D-28 without Intubation. The prescription of corticosteroids therapy at admission was defined as a daily dose ≥ 0.5 mg/kg of prednisone or equivalent during the first 24 hours after admission in ICU.*

***Abbreviations:*** *csHR = cause-specific Hazard Ratio; BMI = Body Mass Index; SOFA Score = Sequential Organ Failure Assessment Score; COPD = Chronic Obstructive Pulmonary Disease, ICU = Intensive Care Unit. NV-ICU-AP= Non-ventilator-associated ICU-acquired pneumonia. MDR= Multi Drug Resistance.*

**Supplemental Table 15. Characteristics at baseline and the day before intubation for patients with non-ventilator-associated ICU-acquired pneumonia (NV-ICU-AP) requiring intubation (N=31).**

|  | At admission (n=32) | Day before intubation (n=31) |
| --- | --- | --- |
|  | Median [Q1; Q3] or n (percentage) | Median [Q1; Q3] or n (percentage) |
| **Baseline characteristics** | | |
| Age (years) | 72.3 [66.6; 77.0] | - |
| Male sex, n (%) | 23 (71.9) | - |
| BMI (kg/m^2^) | 23.4 [20.6; 29.3] | - |
| Hospitalisation before ICU admission, n (%) | 15 (46.9) | - |
| Immunodeficiency, n (%) | 5 (15.6) | - |
| **Therapeutic limitation** | | |
| Limitation of therapeutic effort at admission to ICU, n (%) | 3 (9.4) | - |
| **Severity Score** | | |
| SAPS II | 37.0 [29.5; 45.5] | - |
| SOFA score | 5.0 [3.5; 6.0] | 3.0 [2.0; 4.0] |
| **Arterial blood gas** | | |
| Ph | 7.34 [7.28; 7.40] | 7.32 [7.25; 7.40] |
| Pa02 (mmHg) | 71 [55; 99] | 71 [60; 90] |
| PaCO2 (mmHg) | 50 [42; 62.5] | 53 [42; 68.0] |
| HCO3- (mmol/l) | 26 [23; 32] | 25 [21; 33] |
| **Level of consciousness** | | |
| Glasgow Coma Scale | 10.5 [5.0; 15.0] | 15.0 [12.5; 15.0] |
| No decrease in consciousness Day 1- Day 2 (Glasgow Coma Scale = 15) | 10 (31.2) | - |
| Glasgow Coma Scale < 8 | 11 (34.4) | 0 |
| **Indication for the use of mechanical invasive ventilation** | | |
| Glasgow Coma Scale < 8 | - | 0 |
| Respiratory worsening with ventilatory acidosis (pH ≤ 7.20) | - | 3 (9.7) |
| Refractory hypoxemia (PaO2 < 45 mmHg despite FiO2 > 60%) | - | 1 (2.23) |
| Glasgow Coma Scale < 8 or Respiratory worsening with ventilatory acidosis or Refractory hypoxemia | - | 4 (12.9) |
|  | | |
| **Mortality** | | |
| ICU Mortality rate, n (%) | 15 (46.9) | |
| Hospital Mortality rate, n (%) | 17 (53.1) | |
| Mortality at Day 28, n (%) | 10 (31.2) | |
| **Invasive mechanical ventilation** | | |
| Length of Invasive mechanical ventilation (days) | 13.0 [8.5; 26.0] | |

***Notes:*** *Indications for mechanical ventilation are defined as i) neurological deterioration with coma (Glasgow Coma Scale < 8); ii) respiratory deterioration with ventilatory acidosis (pH ≤ 7.20); iii) refractory hypoxaemia with PaO2 < 45 mmHg despite high inspired fraction of oxygen (FiO2) > 60%. All these criteria are similar to those previously published in studies using NIV and correspond to current daily clinical practice in the ICU (Davidson et al. Thorax. april 2016;71 Suppl 2:ii1- 35; Leuppi et al. JAMA. 5 june 2013;309(21):2223- 31). One patient had missing arterial blood gas data the day before intubation.*

***Abbreviations:*** *ICU = Intensive Care Unit; BMI = Body Mass Index; SAPS II = Simplified Acute Physiology Score II; SOFA Score = Sequential Organ Failure Assessment Score; NV-ICU-AP = Non-ventilator-associated Intensive Care Unit Acquired Pneumonia; Pa02: Partial pressure of oxygen. Pa02: Partial pressure of carbon dioxide; SOFA: Sequential Organ Failure Assessment;* *FiO2: Fraction of inspired oxygen.*

**OUTCOMEREA NETWORK**

***Scientific Committee*:** Jean-François Timsit (Medical and Infectious Diseases ICU, Bichat-Claude Bernard Hospital, Paris, France; UMR 1137 Inserm –Paris Diderot university IAME, F75018, Paris); Elie Azoulay (Medical ICU, Saint Louis Hospital, Paris, France); Maïté Garrouste-Orgeas (Paliative care, Institut Franco Britanique, Paris, France); Jean-Ralph Zahar (Infection Control Unit, Angers Hospital, Angers, France); Bruno Mourvillier (Medical ICU, CHU Reims,France); Michael Darmon (Medical ICU, APHP Saint Louis hospital Paris, France).

***Biostatistical and Information System Expertise*:** Jean-Francois Timsit (Medical and Infectious Diseases ICU, Bichat-Claude Bernard Hospital, Paris, France; UMR 1137 Inserm –Paris Diderot university IAME, F75018, Paris); Corinne Alberti (Medical Computer Sciences and Biostatistics Department, Robert Debré Hospital, Paris, France); Stephane Ruckly (OUTCOMEREA organization and Inserm UMR 1137 IAME, F75018, Paris); Sébastien Bailly (Grenoble Alpes University, INSERM 1300, HP2, Grenoble, France) and Aurélien Vannieuwenhuyze (Tourcoing, France).

***Investigators of the OUTCOMEREA Database*:** Christophe Adrie (ICU, CH Melun, and Physiology, Cochin Hospital, Paris, France); Carole Agasse (Medical ICU, University Hospital Nantes, France); Bernard Allaouchiche (ICU, Hospices civils de Lyon, Lyon sud, Lyon, France); Olivier Andremont (ICU, Bichat Hospital, Paris, France); Pascal Andreu (CHU Dijon, Dijon, France); Laurent Argaud (Medical ICU, Hospices Civils de Lyon, Lyon, France); Claire Ara-Somohano (Medical ICU, University Hospital, Grenoble, France); Elie Azoulay (Medical ICU, Saint Louis Hospital, Paris, France); Francois Barbier (medical-surgical ICU, Orléans, France), Jean-Pierre Bedos (ICU, Versailles Hospital, Versailles, France); Thomas Baudry (Medial ICU, Edouard Heriot hospital, Lyon France), Julien Bohé (ICU, Hôpital Pierre Benite, Lyon France), Lila Bouadma (ICU, Bichat Hospital, Paris, France); Jeremy Bourenne (Réanimation des urgences, Timone-2; APHM, Marseille, France); Noel Brule (medical ICU, university hospital Nantes, France); Frank Chemouni (Grand Hôpital de l’Est Francilien Site Marne La vallée ; Polyvalent ICU, Jossigny Polyvalent ICU) ; Julien Carvelli (Réanimation des urgences, Timone-2; APHM, Marseille, France); Elisabeth Coupez (ICU, G Montpied Hospital, Clermont-Ferrand, France); Martin Cour Medial ICU, Edouard Heriot hospital, Lyon France), Michael Darmon (ICU, APHP St louis, Paris France); Claire Dupuis (ICU, G Montpied Hospital, Clermont-Ferrand, France), Etienne de Montmollin (ICU, Bichat Hospital, Paris, France), Loa Dopeux (ICU, G Montpied Hospital, Clermont-Ferrand, France); Anne-Sylvie Dumenil (Antoine Béclère Hospital, Clamart, France); Claire Dupuis (Bichat hospital and UMR 1137 Inserm –Paris Diderot university IAME, F75018, Paris, France), Jean-Marc Forel (AP HM, Medical ICU, Hôpital Nord Marseille), Marc Gainnier (Réanimation des urgences, Timone-2; APHM, Marseille, France), Charlotte Garret (Medical ICU, university hospital Nantes, France); Dany Goldgran-Tonedano ( CH le Raincy-Montfermeil; France); Steven Grangé (ICU, CHU Rouen, France), Antoine Gros (ICU, Versailles Hospital, Versailles, France), Hédia Hammed (CH le Raincy-Montfermeil) ; Akim Haouache (Surgical ICU, H Mondor Hospital, Créteil, France); Tarik Hissem (ICU, Eaubonne, France), Vivien Hong Tuan Ha (ICU, CH Meaux, France); Sébastien Jochmans (ICU, CH Melun); Jean-Baptiste Joffredo (ICU, G Montpied Hospital, Clermont-Ferrand, France); Hatem Kallel (ICU, Cayenne General Hospital, Cayenne, France); Guillaume Lacave (ICU, Versailles Hospital, Versailles, France), Virgine Laurent (ICU, Versailles Hospital, Versailles, France), Alexandre Lautrette (ICU, G Montpied Hospital, Clermont-Ferrand, France); Clément Le bihan (ICU, Bichat Hospital, Paris, France), Virgine Lemiale (Medical ICU, Saint Louis Hospital, Paris, France); David Luis (Médecine intensive et réanimation, CH Simone Veil, Beauvais, France), Guillaume Marcotte (Surgical ICU, Hospices Civils de Lyon, Lyon, France); Jordane Lebut (ICU, Bichat Hospital, Paris, France); Bruno Mourvillier (ICU, Bichat Hospital, Paris, France); Benoît Misset (ICU, Saint-Joseph Hospital, Paris, France); Bruno Mourvillier (ICU, Medical ICU, Reims France); Mathild Neuville (ICU, Foch Hospital, Paris, France) ; Laurent Nicolet (Medical ICU, university hospital Nantes, France); Johanna Oziel (Medico-surgical ICU, hôpital Avicenne APHP, Bobigny, France), Laurent Papazian (Hôpital Nord, Marseille, France), Juliette Patrier (ICU, Bichat Hospital, Paris, France), Benjamin Planquette (pulmonology ICU, George Pompidou hospital Hospital, Paris, France); Aguila Radjou (ICU, Bichat Hospital, Paris, France), Marie Simon (Medial ICU, Edouard Heriot hospital, Lyon France), Romain Sonneville (ICU, Bichat Hospital, Paris, France), Jean Reignier (Medical ICU, university hospital Nantes, France); Bertrand Souweine (ICU, G Montpied Hospital, Clermont-Ferrand, France); Carole Schwebel (ICU, A Michallon Hospital, Grenoble, France); Shidasp Siami (ICU, Eaubonne, France); Romain Sonneville (ICU, Bichat Hospital, Paris, France); Nicolas Terzi (ICU, A Michallon Hospital, Grenoble, France ) ; Gilles Troché (ICU, Versailles Hospital, Versailles, France); Fabrice Thiollieres (ICU, Hospices civils de lyon, Lyon sud, Lyon, France) ; Guillaume Thierry (ICU, St Etienne, France); Guillaume Van Der Meersch (Medical Surgical ICU, university hospital Avicenne), Marion Venot (Medical ICU, Saint Louis Hospital, Paris, France); Florent Wallet (ICU, Hospices civils de lyon, Lyon sud, Lyon, France) : Sondes Yaacoubi (CH le Raincy-Montfermeil); Olivier Zambon (medical ICU, university hospital Nantes, France); Jonathan Zarka (Réanimation polyvalente, centre hospitalier de Marne la Vallee, France).

***Study Monitors****: Mireille Adda, Vanessa Vindrieux, Marion Provent, Sylvie de la Salle, Pauline Enguerrand, Vincent Gobert, Stéphane Guessens, Helene Merle, Nadira Kaddour, Boris Berthe, Samir Bekkhouche, Kaouttar Mellouk, Mélaine Lebrazic, Carole Ouisse, Diane Maugars, Christelle Aparicio, Igor Theodose, Manal Nouacer, Veronique Deiler, Fariza Lamara, Myriam Moussa, Atika Mouaci, Nassima Viguier.*
